# Upregulated PD-1 signaling antagonizes glomerular health in aged kidneys and disease

**DOI:** 10.1172/JCI156250

**Published:** 2022-08-15

**Authors:** Jeffrey W. Pippin, Natalya Kaverina, Yuliang Wang, Diana G. Eng, Yuting Zeng, Uyen Tran, Carol J. Loretz, Anthony Chang, Shreeram Akilesh, Chetan Poudel, Hannah S. Perry, Christopher O’Connor, Joshua C. Vaughan, Markus Bitzer, Oliver Wessely, Stuart J. Shankland

**Affiliations:** 1 Division of Nephrology,; 2 Paul G. Allen School of Computer Science and Engineering, and; 3 Department of Chemistry, University of Washington, Seattle, Washington, USA.; 4 Lerner Research Institute, Cleveland Clinic Foundation, Cleveland, Ohio, USA.; 5 Department of Pathology, University of Chicago, Chicago, Illinois, USA.; 6 Department of Pathology, University of Washington, Seattle, Washington, USA.; 7 Division of Nephrology, University of Michigan, Ann Arbor, Michigan, USA.; 8 Department of Physiology and Biophysics and; 9 Institute for Stem Cell and Regenerative Medicine, University of Washington, Seattle, Washington, USA.

**Keywords:** Aging, Chronic kidney disease, Expression profiling

## Abstract

With an aging population, kidney health becomes an important medical and socioeconomic factor. Kidney aging mechanisms are not well understood. We previously showed that podocytes isolated from aged mice exhibit increased expression of programmed cell death protein 1 (PD-1) surface receptor and its 2 ligands (PD-L1 and PD-L2). *PDCD1* transcript increased with age in microdissected human glomeruli, which correlated with lower estimated glomerular filtration rate and higher segmental glomerulosclerosis and vascular arterial intima-to-lumen ratio. In vitro studies in podocytes demonstrated a critical role for PD-1 signaling in cell survival and in the induction of a senescence-associated secretory phenotype. To prove PD-1 signaling was critical to podocyte aging, aged mice were injected with anti–PD-1 antibody. Treatment significantly improved the aging phenotype in both kidney and liver. In the glomerulus, it increased the life span of podocytes, but not that of parietal epithelial, mesangial, or endothelial cells. Transcriptomic and immunohistochemistry studies demonstrated that anti–PD-1 antibody treatment improved the health span of podocytes. Administering the same anti–PD-1 antibody to young mice with experimental focal segmental glomerulosclerosis (FSGS) lowered proteinuria and improved podocyte number. These results suggest a critical contribution of increased PD-1 signaling toward both kidney and liver aging and in FSGS.

## Introduction

The US population is aging, and the number of Americans aged 65 and older will more than double over the next 40 years (US Census Bureau); Eurostat forecasts that 28% of Europeans will be older than 65 years by 2060. As life expectancy increases, the impact of advanced age on kidney health and function is becoming an increasingly important medical and socioeconomic factor. Glomerular filtration rate (GFR) declines after age 40 by 0.8%–1.0% per year (1, 2), and kidneys from healthy 70- to 75-year-old individuals have 48% fewer intact nephrons compared with those from 19- to 29-year-old individuals (3), which is consistent with an estimated annual loss of 6000–6500 nephrons after age 30 (3–5).

The characteristic physiologic, histologic, and molecular changes to the aged kidney have been well described and reviewed elsewhere (5–10). At the cellular level, age-dependent glomerulosclerosis and accompanying decline in GFR are paralleled by changes in number, structure, and function to all 4 resident glomerular cell types (podocytes, mesangial cells, endothelial cells, and parietal epithelial cells) (11–13). By now, age-dependent glomerulosclerosis is regarded as a podocyte disorder (14). Studies in animal models demonstrated that podocyte loss causes glomerulosclerosis in direct proportion to the degree of depletion (15–17). Similarly, upon aging, podocyte numbers and density decrease in rats and mice (11, 18–21). In humans, Hodgin, Wiggins, and colleagues (22) showed that the podocyte reserve dropped about 0.9% annually from more than 300 per 100 cm^3^ in young kidneys to less than 100 per 100 cm^3^ by 70–80 years of age. In fact, increased age is independently associated with both absolute and relative podocyte depletion (23).

While a recent study demonstrated a critical role of GSK3b as a mechanism involved in podocyte aging in general (24), the causes of podocyte changes in aged kidneys are still not completely understood (25). To gain insights into candidate mechanisms at the transcriptomic level, we recently performed bulk RNA-Seq comparing podocytes from 2- to 3-month-old mice (corresponding to ~20-year-old humans) versus 24-month-old mice (corresponding to humans older than ~70 years) (26). We discovered a statistically significant increase in the expression of programmed cell death RNA transcript 1 (PD-1; synonyms PDCD1 and CD279), programmed cell death 1 ligand 1 (PD-L1; synonyms CD274 and B7-H1), and programmed cell death 1 ligand 2 (PD-L2; synonyms CD273 and B7-DC) in aged podocytes compared with young podocytes (26). The 288–amino acid, type I membrane protein PD-1 is typically expressed on immature immune cells and is bound by the 2 ligands of the B7 family, PD-L1 and PD-L2 (27, 28). In addition to being expressed on circulating immune cells, PD-1, PD-L1, and PD-L2 are also expressed in various cancers and many other cell types (29). Clinical studies have shown that blocking the PD-1/PD-1 ligand signaling pathway with checkpoint inhibitors improves outcomes in many forms of cancer (30, 31).

The functional consequences of increased levels of PD-1 and its ligands in aged podocytes are unknown. Based on the increased mRNA levels of PD-1 and its ligands in aged podocytes, we hypothesized that reducing PD-1/PD-1 ligand signaling in the aged kidney would improve podocyte health. To this end, we performed both in vitro and in vivo experiments to systemically determine the impact of PD-1 signaling on glomerular health during aging.

## Results

### PD-1 and PD-1 ligands increase in aged mice and human kidneys.

**Figure 1:**
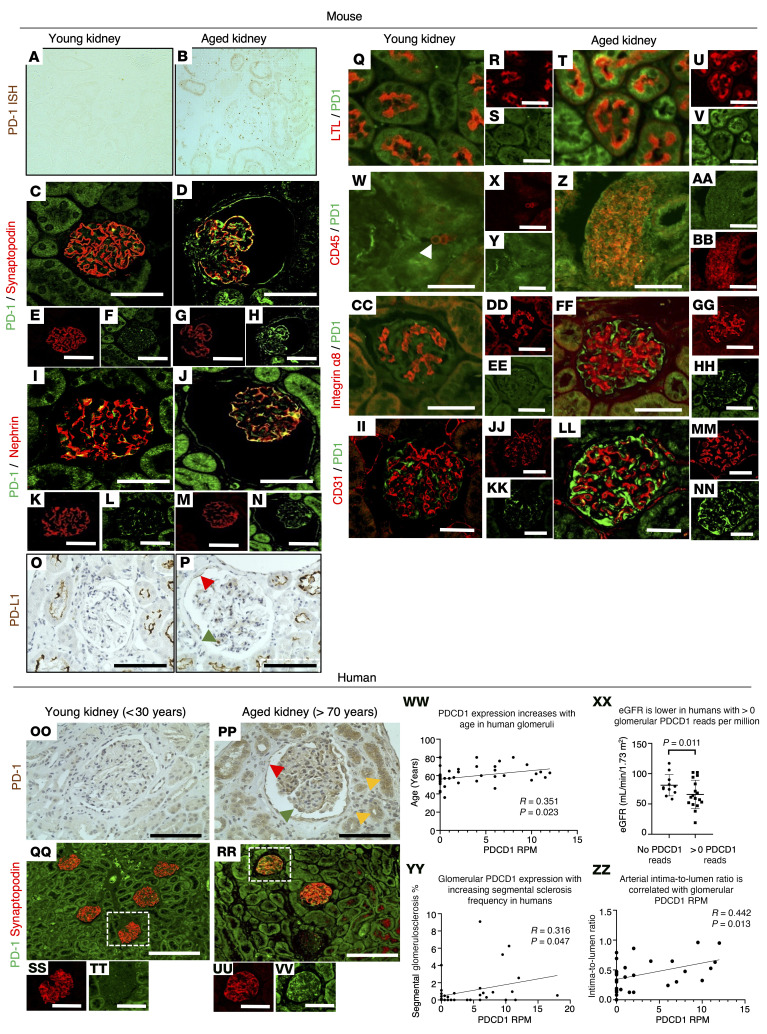
Podocyte PD-1 immunostaining and transcripts. (**A**–**NN**) Mouse kidney. (**A** and **B**) In situ hybridization shows that compared with young kidney (**A**), PD-1 transcript (brown) increases in aged kidney (**B**). (**C**–**H**) PD-1 (green) and synaptopodin (red) staining shows that PD-1 in mouse glomerulus (**C**) merges with synaptopodin-positive podocytes in aged kidney (**D**, yellow). PD-1 stains PECs along Bowman’s capsule (**D**, green). (**E**–**H**) Fluorescence channels from **C** and **D**. (**I**–**N**) PD-1 (green) and nephrin (red) staining in young glomeruli (**I**) merges with nephrin in aged kidney (**J**, yellow). PD-1 increased in PECs and proximal tubular epithelial cells (**J**, green). (**K**–**N**) Single channels from **I** and **J**. (**O** and **P**) PD-L1 (brown) in young mouse kidney (**O**) is detected in a podocyte (green arrowhead), PECs (red arrowhead), and proximal tubules in the aged mouse (**P**). (**Q**–**V**) Lotus tetragonolobus lectin (LTL) (red) stains proximal epithelial cells and merges with PD-1 (green) in aged kidney (**T**). (**W**–**BB**) CD45^+^ interstitial lymphocytes (red) merge with PD-1 (green) in aged kidney. (**CC**–**HH**) PD-1 (green) does not merge with the mesangial cell marker α_8_ integrin (red). (**II**–**NN**) PD-1 (green) does not merge with the endothelial cell marker CD31 (red). (**OO**–**VV**) Human kidney. (**OO** and **PP**) PD-1 (brown) in young human kidney (**OO**) increases in podocytes (green arrowhead), PECs (red arrowhead), and tubular epithelial cells (orange arrowheads) in aged human kidney (**PP**). (**QQ**–**VV**) PD-1 (green) and synaptopodin (red) in young human glomerulus merges with synaptopodin (yellow) in aged human glomerulus. (**WW**–**ZZ**) *PDCD1* transcripts from microdissected human glomeruli. Expression of *PDCD1* (corresponding to human PD-1) increased with age (**WW**), accompanied by lower eGFR (**XX**), higher glomerulosclerosis (**YY**), and vascular injury (**ZZ**). Scale bars: 25 μm (**C**–**NN**), 100 μm (**OO**, **PP**, and **SS**–**VV**), and 200 μm (**QQ** and **RR**). Statistical analysis was performed by *t* test, χ^2^ test, and quasi-Poisson regression modeling.

Our recently published mRNA-Seq data showed a 17.4-fold increase in transcript levels for PD-1, a 1.4-fold increase in PD-L1, and a 2.3-fold increase in PD-L2 in podocytes from aged mice compared with young mice (26). This was confirmed in the current study in an independent cohort of young (*n =* 16) and aged (*n =* 15) mice, in which mRNA expression was assayed by quantitative PCR performed on podocyte fractions sorted by magnetic-activated cell sorting (MACS; Miltenyi Biotec). *PD-1* increased 6.7-fold (1.6 ± 0.2 young vs. 10.1 ± 1.2 aged, *P <* 0.0001), *PD-L1* increased 2.3-fold (3.6 ± 0.3 young vs. 8.4 ± 0.8 aged, *P <* 0.0001), and *PD-L2* increased 17.3-fold (12.8 ± 2.9 young vs. 221.4 ± 35.7 aged, *P <* 0.0001) in aged podocytes. In situ hybridization confirmed the increase in *PD-1* mRNA in aged glomeruli (Figure 1, A and B).

Immunofluorescent staining substantiated these findings at the protein level, showing that PD-1 was barely detected in young mouse kidneys, but was markedly elevated in aged kidneys (Figure 1, C–N). Coimmunostaining of anti–PD-1 antibody with cell type–specific markers showed that in aged mice the increased PD-1 protein colocalized with synaptopodin- and nephrin-positive podocytes and localized to parietal epithelial cells (PECs) lining Bowman’s capsule (Figure 1, D and J). This was accompanied by increased PD-L1 staining (Figure 1, O and P). In addition, lotus tetragonolobus lectin–positive (LTL-positive) proximal tubular epithelial cells (Figure 1, Q–V) and interstitial CD45-positive lymphocytes (Figure 1, W–BB) were PD-1 positive, while α_8_ integrin–positive mesangial cells (Figure 1, CC–HH) and CD31-positive glomerular endothelial cells (Figure 1, II–NN) were PD-1 negative. The same staining pattern was observed in human kidneys, where PD-1 immunostaining was also increased in aged podocytes, PECs, and tubular epithelial cells, but not in young human kidneys (Figure 1, OO–VV).

To examine potential functional consequences of PD-1 expression in aged humans, we analyzed transcriptomic data from microdissected glomeruli from aged human kidneys for correlations between *PDCD1* (gene name for human PD-1) and clinical parameters for glomerular aging and function. *PDCD1* expression in humans increased with age (*P <* 0.023, *R* = 0.351; Figure 1WW). Importantly, the increased *PDCD1* transcript levels were accompanied by a lower estimated GFR (eGFR) (*P =* 0.011; Figure 1XX), and correlated with increased segmental glomerulosclerosis (*P =* 0.047, *R* = 0.316; Figure 1YY) as well as reduced arterial intima-to-lumen ratio (*P =* 0.013, *R* = 0.0442; Figure 1ZZ), a measure of vascular injury.

These results show that the PD-1 pathway increases in aged mouse and human glomeruli, and correlates with glomerular scarring and vascular damage and declining kidney function.

### Overexpression of PD-1 is sufficient to induce death in cultured podocytes.

**Figure 2:**
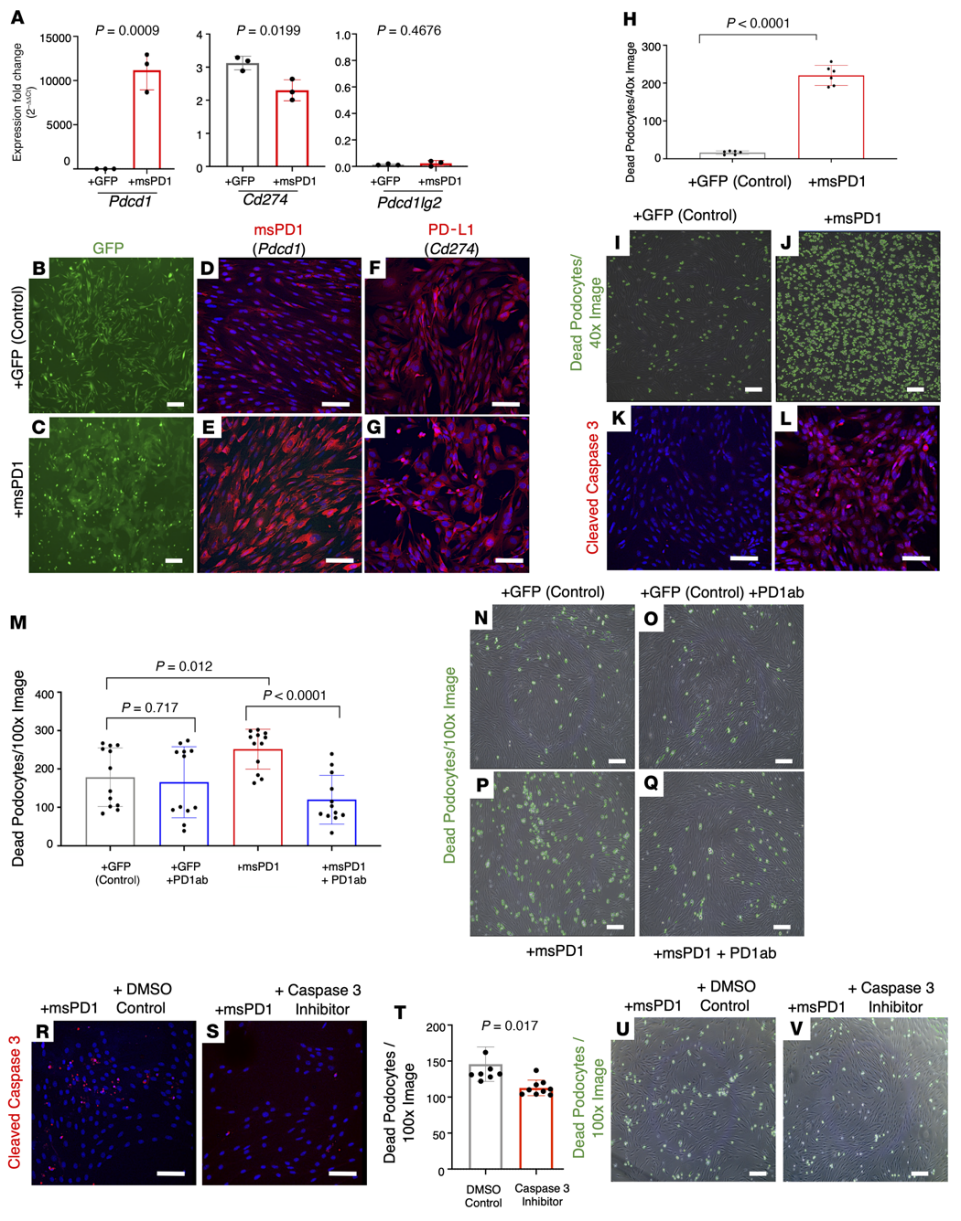
Overexpression of PD-1 induces podocyte death in cell culture. (**A**–**G**) Following overexpression of PD-1 using a lentiviral expression vector (msPD1, red bars) in immortalized mouse podocytes, mRNA levels increased significantly for *Pdcd1* (PD-1) in comparison with GFP control–infected podocytes, without changes to *Cd274* or *Pdcd1lg2*. GFP expression (green) of the GFP control (**B**) and PD-1–overexpressing (**C**) lentiviral vectors confirms efficient transfection. Immunocytochemistry for PD-1 protein (red) in GFP control–infected podocytes (**D**) was increased in msPD1-overexpressing podocytes (**E**). DAPI stains nuclei blue. Staining for PD-L1 (red) was not different between GFP control–infected (**F**) and PD-1–overexpressing (**G**) podocytes. (**H**–**L**) Overexpression of PD-1 (red bar) increased podocyte death in comparison with GFP control–infected podocytes (gray bar). Representative images of dead podocytes (**I** and **J**). Cleaved caspase-3 staining (red) was barely detected in GFP control–infected podocytes (**K**) but was markedly increased in PD-1–overexpressing podocytes (**L**). Nuclei were counterstained with DAPI (blue). (**M**–**Q**) Application of aPD1ab (blue bar, second column) did not impact cell death in GFP control–infected podocytes (gray bar). The increased podocyte death induced by overexpression of PD-1 (red bar) was reduced when anti–PD-1 antibody was applied (blue bar, fourth column). (**N**–**Q**) Representative images of dead podocytes encircled with green annotations used for **M**. (**R** and **S**) Immunocytochemistry for cleaved caspase-3 (red) was increased in PD-1–overexpressing podocytes treated with DMSO (**R**) but was markedly decreased following treatment with the caspase-3–specific inhibitor Z-DEVD-FMK (**S**). (**T**–**V**) Treatment of PD-1–overexpressing podocytes with the caspase-3 inhibitor significantly decreased podocyte death (red bar) compared with the DMSO control (gray bar). (**U** and **V**) Representative images of dead podocytes encircled with green annotations in DMSO-treated (**U**) and caspase-3 inhibitor–treated (**V**) PD-1–overexpressing podocytes. Statistical analysis was performed by *t* test. Scale bars represent 100 μm.

To address whether increased PD-1 signaling has a biological role, we used immortalized mouse podocytes (32–34), which endogenously express one of the PD-1 ligands, PD-L1, yet had very low levels of PD-1 itself and its other ligand, PD-L2 (Figure 2, A, D, and F). Ectopic expression resulted in highly elevated PD-1 levels but did not impact the expression of either ligand (Figure 2, A, E, and G). The presence of green fluorescent protein (GFP) confirmed the transduction of the control and PD-1–overexpressing lentiviral vectors (Figure 2, B and C). Overexpression of PD-1 had dramatic effects on podocyte survival, inducing apoptosis as assessed by cleaved caspase-3 staining and dead cell quantification (Figure 2, H–L). To verify that this was indeed due to induced PD-1 signaling, we used a neutralizing anti–PD-1 antibody (referred to as aPD1ab). Treating PD-1–overexpressing podocytes with aPD1ab restored the levels of podocyte death to the levels observed for the GFP vector control podocytes (Figure 2, M–Q). Exposing PD-1–overexpressing podocytes to a caspase-3 inhibitor reduced cleaved caspase-3 levels (Figure 2, R and S), which in turn reduced cell death (Figure 2, T–V). Thus, in vitro experiments in immortalized podocytes support a critical role for PD-1 signaling through caspase-3 in podocyte apoptosis.

### Impact of PD-1 inhibition on glomerular aging in mice.

To test whether the upregulation of PD-1/PD-L1/PD-L2 is biologically relevant for aged podocytes in vivo, we inhibited PD-1 signaling using a neutralizing rat anti–mouse PD-1 antibody. Twenty-one-month-old mice were randomized and injected intraperitoneally with aPD1ab or the control IgG2a antibody (referred to as IgG2a) once weekly for a total of 8 weeks (Supplemental Figure 1A; supplemental material available online with this article; https://doi.org/10.1172/JCI156250DS1). Uninjected, 4-month-old mice were used as young age controls. Treatments had no major impact on mortality, body weight, or kidney function as measured by urinary albumin/creatinine ratios, blood urea nitrogen, serum creatinine, and blood soluble urokinase plasminogen activator receptor (Supplemental Figure 1, B–F). Organs (blood, kidney, liver, and spleen) were extracted and processed for immunofluorescence/histology. To detect the distribution of the injected aPD1ab or control IgG2a, immunofluorescence staining with an anti-rat IgG2a antibody confirmed that the aPD1ab reached the glomerular and tubular epithelium and bound in a similar pattern to PD-1 expression. Control IgG2a antibody did not bind. aPD1ab deposition also reflected PD-1 expression in the liver (Supplemental Figure 1G). To specifically assess the effect of aPD1ab on the podocyte transcriptome, kidneys were digested and separated into podocyte and non-podocyte cell fractions by MACS.

**Figure 3:**
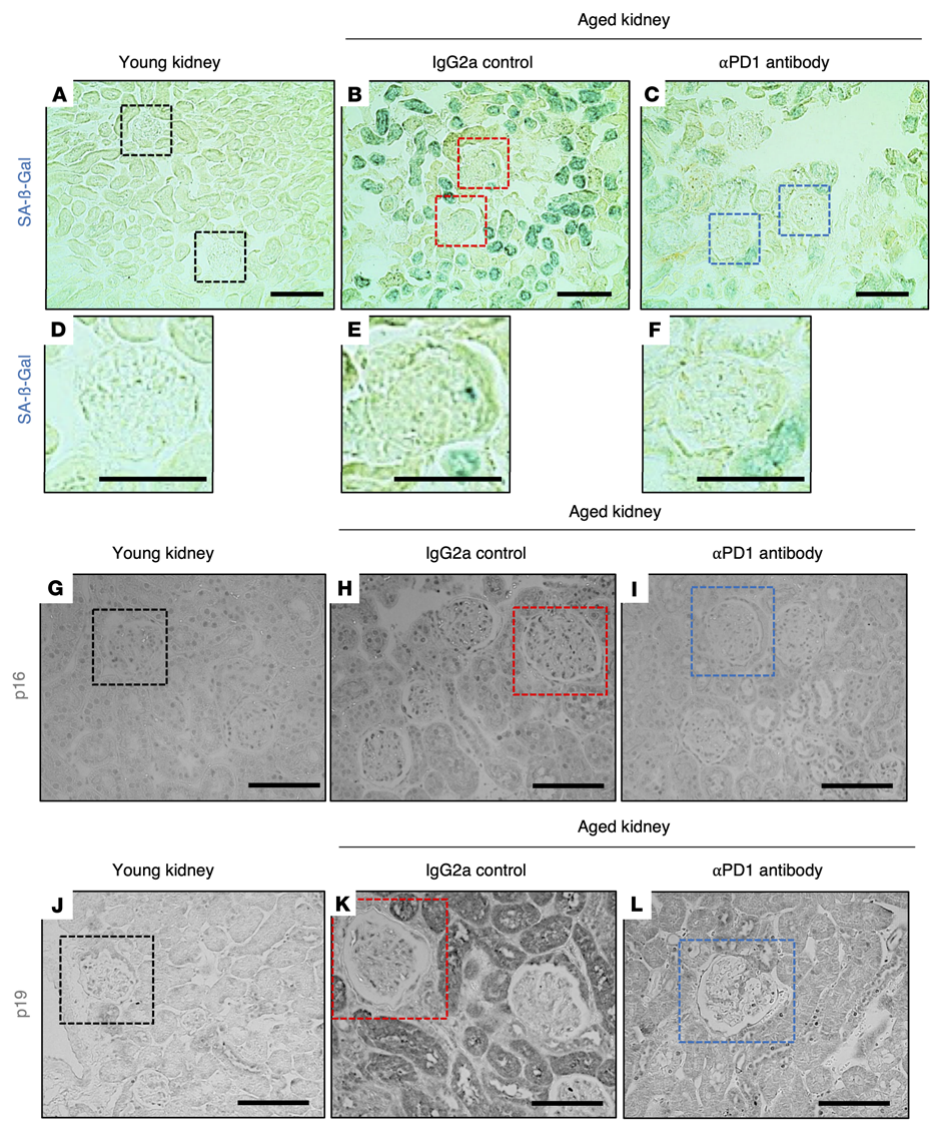
Podocyte senescence. (**A**–**F**) Representative images of SA-β-gal staining (blue). SA-β-gal was barely detected in young kidneys (**A** and **D**) but increased in glomeruli (red boxes) and tubular epithelial cells in IgG2a-injected aged mice (**B** and **E**) and was lowered by aPD1ab in glomeruli (blue boxes) and tubules (**C** and **F**). (**G**–**I**) p16 staining (black) was occasionally detected in glomeruli and tubular epithelial cells of young kidneys (**G**). It was increased in glomerular (red box) and tubular epithelial cells in IgG2a-injected mice (**H**) but was lower in aPD1ab-injected mice (**I**). (**J**–**L**) p19 staining (black) was barely detected in young kidneys (**J**) but was increased in glomerular (red box) and tubular epithelial cells in IgG2a-injected mice (**K**) and was lower in aPD1ab-injected mice (**L**). Scale bars: 50 μm.

We next addressed whether the aPD1ab treatment impacted kidney aging. As expected, when compared with their young counterparts (Figure 3, A and D), staining for senescence-associated β-galactosidase (SA-β-gal) (9) was higher in both glomerular and tubular epithelial cells in aged mice (Figure 3, B and E). Treatment with aPD1ab caused a reduction in both glomerular and tubular SA-β-gal (Figure 3, C and F). The same pattern was observed in immunostainings for the senescence proteins p16 and p19 (9), where aPD1ab-injected mice exhibited reduced staining compared with age-matched control kidneys (Figure 3, G–L).

**Figure 4:**
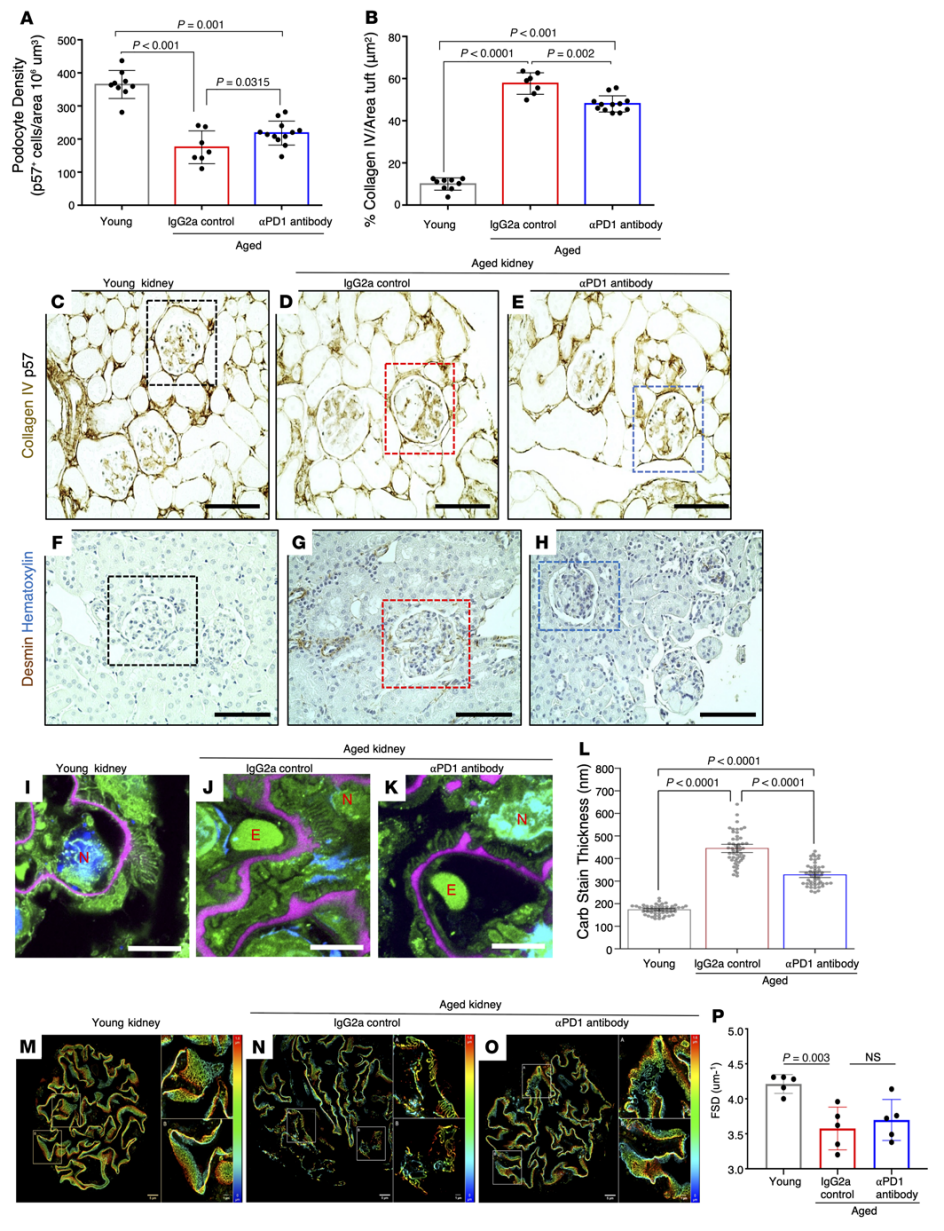
Podocyte density, scarring, stress, and ultrastructure. (**A**–**E**) Podocyte density measured by p57 staining (dark blue, **C**–**E**) and summarized in **A**. Each circle represents an individual mouse. Density was lower in aged IgG2a-injected mice compared with young mice and was increased in aged aPD1ab-injected mice. Glomerular scarring was measured by glomerular collagen IV staining (brown, **C**–**E**) and is summarized in **B**. It was higher in IgG2a-injected aged mice compared with young mice and was lowered by aPD1ab. (**F**–**H**) The podocyte stress marker desmin (brown) was increased in aged IgG2a-injected mice compared with young and was lower in aged aPD1ab mice. (**I**–**L**) The filtration barrier ultrastructure was assessed by expansion microscopy of FLARE-labeled glomeruli, which demonstrated that glomerular basement membrane (GBM) thickness (pink) was significantly increased in aged IgG2a-injected mice (**J**) compared with young mice (**I**) and reduced in aged aPD1ab mice (**K**). Representative images are shown in **I**–**K**, and GBM thickness is quantified in **L**. N, nuclei; E, erythrocytes. (**M**–**P**) Podocyte ultrastructure was characterized by the podocyte exact morphology measurement procedure (PEMP). Representative images are shown in **M**–**O**, and filtration slit density (FSD) is quantified in **P**. This analysis shows a significant decrease in FSD in aged IgG2a-injected mice compared with young mice (**M**, **N**, and **P**). Elevation of FSD was observed in aged aPD1ab mice but did not reach significance (**O** and **P**). Scale bars: 5 μm (**M**–**O**), 10 μm (**I**–**K**), and 50 μm (**C**–**H**). Statistical analysis was performed by 1-way ANOVA.

A second hallmark of glomerular aging is the decrease in the podocyte’s life span, measured by a decrease in podocyte density (22). Measuring podocyte density using a computer-assisted machine learning approach showed a decrease in aged (i.e., IgG2a control–injected) versus young mice (*P <* 0.001), which was partially restored in the age-matched mice injected with aPD1ab (*P =* 0.0315) (Figure 4A). This was accompanied by changes in glomerular collagen IV (21) (Figure 4, B–E) and the stress marker desmin (14, 18) (Figure 4, F–H). Both were increased in aged mice and reduced upon treatment with aPD1ab. Finally, to assess podocyte ultrastructure, expansion microscopy of glomeruli labeled with fluorescent labeling of abundant reactive entities (FLARE) demonstrated an age-dependent increase in the thickness of the glomerular basement membrane, which was significantly reduced upon injection of aPD1ab (Figure 4, I–L, and Supplemental Videos 1–3). To assess filtration slit density, podocyte exact morphology measurement procedure (PEMP) (35) was performed. Filtration slit density significantly decreased during aging and trended toward improvement following anti–PD-1 treatment but did not reach statistical significance (Figure 4, M–P).

**Figure 5:**
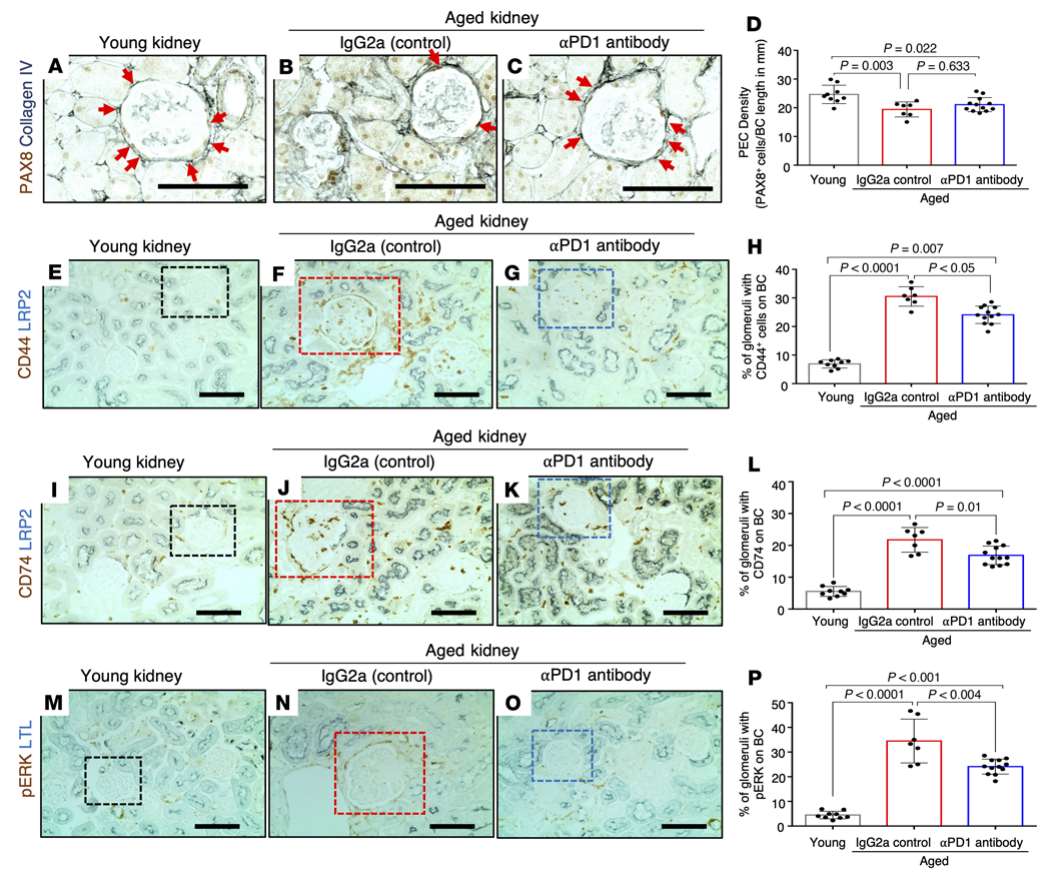
Parietal epithelial cell changes. (**A**–**D**) Representative images of immunoperoxidase staining for the PEC marker PAX8 (brown) and collagen IV (blue, outlines Bowman’s capsule [BC]) (**A**–**C**) and quantification thereof (**D**). PEC density was lower in aged IgG2a-injected mice (red bar) compared with young mice (gray bar) but did not change with aPD1ab treatment (blue bar). (**E**–**P**) Representative images of immunoperoxidase double staining with antibodies against the PEC activation markers CD44 (**E**–**G**, brown), CD74 (**I**–**K**, brown), and p-ERK (**M**–**O**, brown) counterstained with the proximal tubular cell markers LRP2 (**E**–**G** and **I**–**K**, blue) and LTL (**M**–**O**, blue). Quantification (**H**, **L**, and **P**) shows that all 3 PEC activation markers were elevated in aged IgG2a-injected mice (red bars) compared with young mice (gray bars) and lowered by aPD1ab injections (blue bars). Scale bars represent 50 μm. Statistical analysis was performed by *t* test.

We next analyzed the other resident glomerular cell types. PECs marked by the expression of PAX8 were decreased with age, but in contrast to podocytes, their numbers were not restored by aPD1ab treatment (Figure 5, A–D). However, other aspects of PEC aging, including the induction of an activated PEC phenotype as measured by increased expression of the activation markers CD44, CD74, and phosphorylated ERK (p-ERK) and an increase in collagen IV staining along Bowman’s capsule (20, 21), were all significantly increased with age and partially restored in PECs of aged mice injected with aPD1ab (Figure 5, E–P).

The same was observed for glomerular endothelial cells (GENs). GEN density, identified by nuclear staining for the ETS transcription factor ERG (36, 37), was reduced in aged IgG2a control mice (443 ± 10 young vs. 290 ± 19 aged IgG2a control ERG^+^ nuclei × 10^6^ μm^3^, *P <* 0.0001), but was unchanged with aPD1ab (290 ± 19 aged IgG2a control vs. 287 ± 10 aged aPD1ab ERG^+^ nuclei × 10^6^ μm^3^, *P =* 0.902) (Supplemental Figure 2, A–D). Yet their age-dependent increase in the fenestral diaphragm protein plasmalemmal vesicle–associated protein-1 (PV-1), normally absent in healthy GENs and, when present, representing an immature and injured phenotype (38–40), was lowered by administration of aPD1ab (Supplemental Figure 2, E–G). Mesangial cell area, stained by Itga8 (α_8_ integrin), was increased in aged IgG2a control mice compared with young mice, a trend that was also not changed by aPD1ab (Supplemental Figure 2, H–K). Finally, in the tubular compartment, staining for the epithelial cell injury marker KIM-1, and injury transcripts for *Havcr1*, *Lcn1*, and *Vcam1*, were upregulated during aging in control mice, but were lowered in aged aPD1ab mice (Supplemental Figure 2, L–O).

Together, these data demonstrate that interrupting PD-1 signaling can partially reverse the glomerular aging phenotype and improve podocyte life span with respect to cell number, function, and ultrastructure. While it did not impact the numbers or life span of the other glomerular cell types, interfering did have beneficial impacts on the age-dependent activation of PECs and GENs and injury to the tubular epithelium.

### Transcriptomic changes in aged podocytes modified by anti–PD-1 antibody.

To understand the underlying molecular mechanism of PD-1 inhibition, we wondered whether this was due to changes in the mRNA levels of *PD-1* and its ligands. In podocytes, aPD1ab did not alter the mRNA expression of *PD-1* and *PD-L1* measured by quantitative reverse transcriptase PCR, but lowered *PD-L2* (Supplemental Figure 3, A–C). In the non-podocyte fraction, aPD1ab lowered *PD-1*, but did not change levels of *PD-L1* and *PD-L2*. Transcript levels of other members of the B7 ligand family (*CD80*/*B7-1* and *CD86*/*B7-2*) did not change in aged mouse podocytes (not shown). Finally, consistent with at least partial suppression of the PD-1 pathway in vivo, Kyoto Encyclopedia of Genes and Genomes (KEGG) pathway analysis revealed that several downstream targets of the PD-1 signaling pathway were reduced by aPD1ab, including *Cd4*, *Cd28*, *Ptpn6* (Shp1), *Ptpn11* (Shp2), *Pik3cd* (Pi3k), *Rasgfp1*, and *Nfat* (Supplemental Figure 3D).

**Figure 6:**
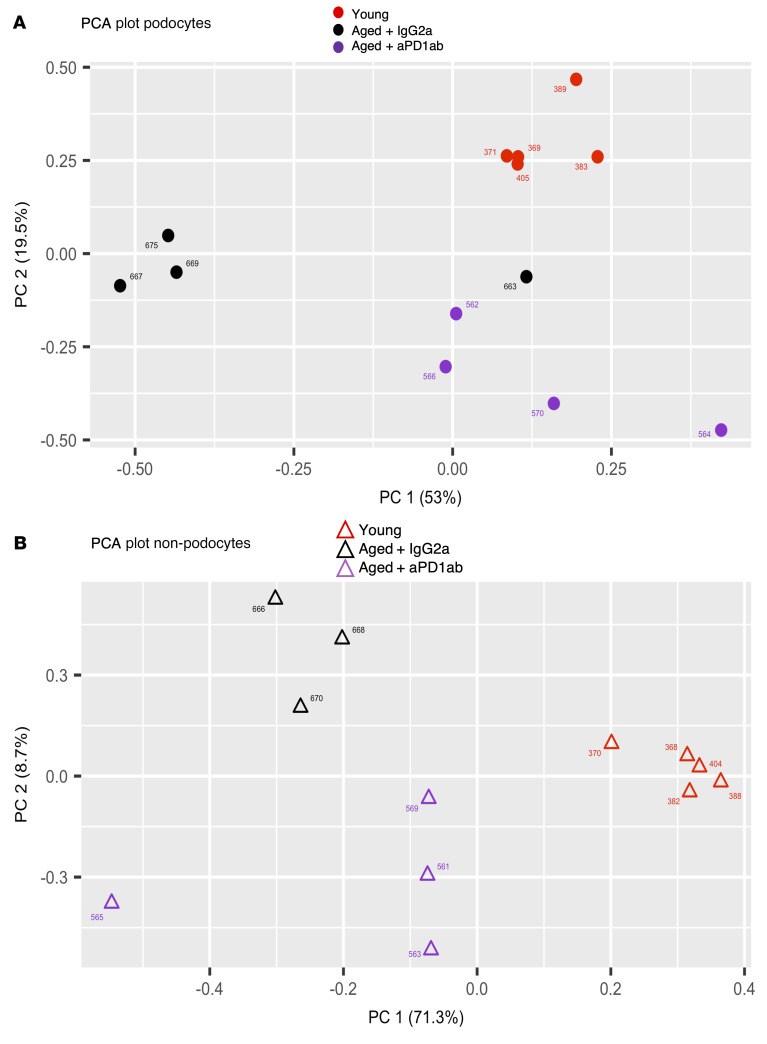
Principal component analysis. Principal component analysis (PCA) of the mRNA-Seq data showed excellent clustering of the individual treatment groups for both podocytes (**A**) and non-podocytes (**B**).

**Figure 7:**
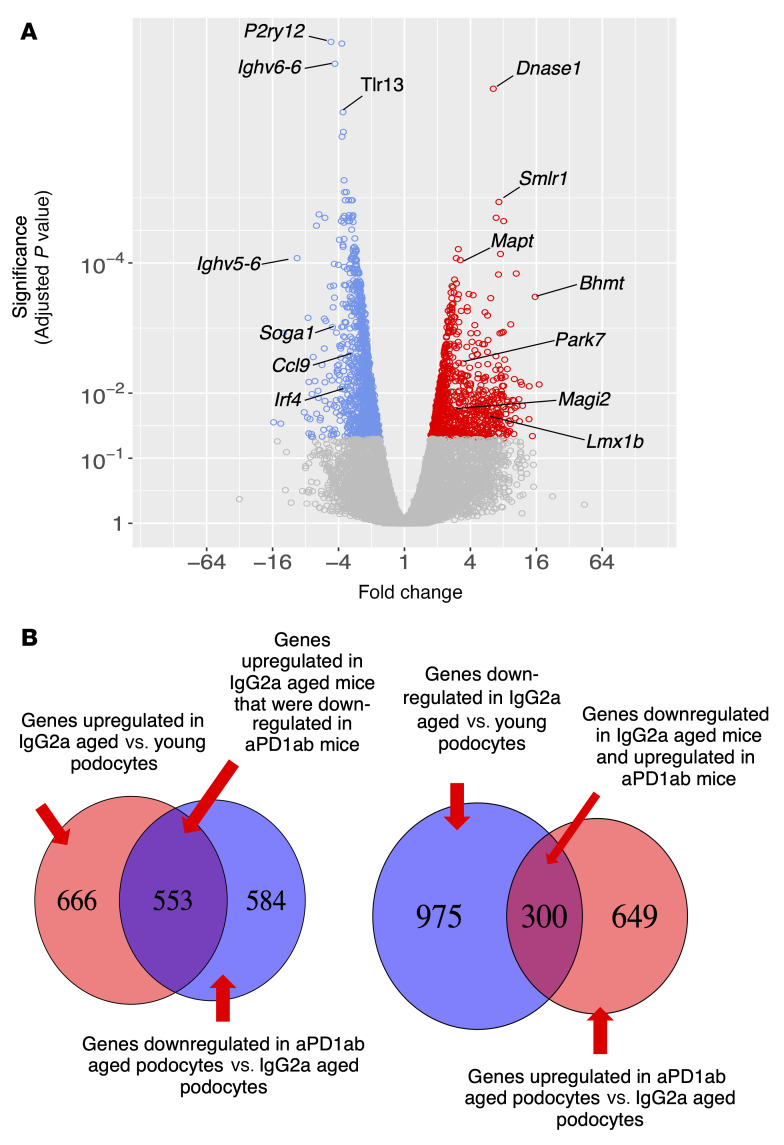
Podocyte transcripts in aged mouse podocytes altered by anti–PD-1 antibody. (**A**) Volcano plot shows the transcripts in aged podocytes that were decreased (blue circles), increased (red circles), or not changed (gray circles) by treatment of mice with aPD1ab for 8 weeks. (**B**) Summary of the number of genes upregulated and downregulated in podocytes from aged control IgG2a mice compared with young mice, and from aged aPD1ab-injected mice compared with age-matched IgG2a-injected mice.

Based on these data and to obtain a better understanding of the mechanism(s) responsible for the reversal of glomerular aging by aPD1ab treatment, we performed mRNA-Seq analyses from podocyte and non-podocyte cell fractions from each individual mouse from each of the groups (young, aged IgG2a-injected control, and aged aPD1ab-injected). Principal component analysis showed excellent clustering of both podocytes and non-podocyte cell fractions in the individual treatment groups (Figure 6, A and B). A total of 1137 genes were downregulated and 949 were upregulated in aged aPD1ab-injected compared with aged IgG2a-injected podocytes (FDR < 0.05 and >2-fold change; Figure 7, A and B). We next analyzed the significantly altered transcripts for their contribution to the aging process. Of the downregulated transcripts, about half of genes (i.e., 553) were also upregulated in aged versus young podocytes (*P =* 0). Similarly, of the upregulated transcripts, about a third (i.e., 300) were downregulated in aged versus young podocytes (*P =* 1 × 10^–32^). Six hundred forty-nine genes were upregulated in aged aPD1ab-injected podocytes compared with aged IgG2-injected podocytes. Together, these data suggest that more than 40% of the genes regulated by PD-1 are part of the natural podocyte aging process.

### Podocyte genes, function, and transcription factors are restored upon anti–PD-1 antibody treatment.

**Figure 8:**
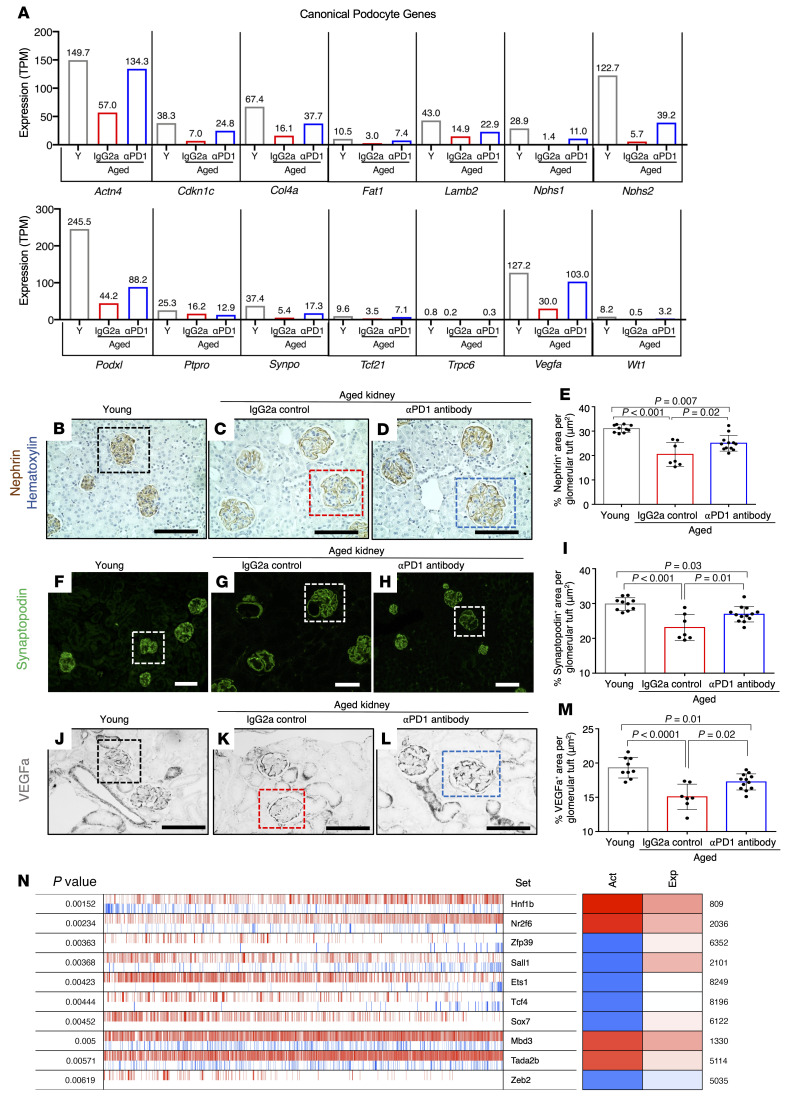
Changes to podocyte canonical genes, proteins, and transcription factors. (**A**) Expression of individual canonical podocyte genes from mRNA-Seq data of young mice (Y, gray bars), aged mice injected with IgG2a (red bars), and aged mice injected with anti–PD-1 antibody (αPD1, blue bars). The levels of all genes were lower in aged IgG2a-injected mice compared with young mice, and all except *Ptpro* were increased in aPD1ab-injected mice. TPM, transcripts per million. (**B**–**M**) Protein validation of select genes by immunostaining. Representative immunoperoxidase staining for nephrin (**B**–**D**, brown), immunofluorescent staining for synaptopodin (**F**–**H**, green), and immunoperoxidase staining for VEGFA (**J**–**L**, black). The box in each panel shows an example of a glomerulus. Bar graphs show the quantification of nephrin (**E**), synaptopodin (**I**), and VEGFA (**M**) staining with each circle representing an individual mouse. Compared with young mice (gray bars), immunostaining was lower for each in aged IgG2a-injected mice (red bars) but was higher in aged aPD1ab-injected mice (blue bars). (**N**) VIPER (virtual inference of protein activity by enriched regulon) analysis of transcription factor activity. The top 10 transcription factors impacted by aPD1ab treatment are shown in the third column, along with their significance (first column), representative activity (second column), conferred activity (fourth column), and expression (fifth column), with red showing increased and blue decreased levels/activities. The conferred activity of *Hnf1b*, *Nr2f6*, *Mbd3*, and *Tada2b* increased, with increased expression. *Zfp39*, *Sall1*, and *Sox7* activity was decreased despite higher expression levels in aPD1ab-injected mice. While the lower activity of *Ets1* and the lower activity of *Tcf4* were independent of their expression levels, the lower activity of *Zeb2* correlated with its lower expression. Scale bars represent 50 μm (**B**–**D** and **J**–**L**) and 100 μm (**F**–**H**). Statistical analysis was performed by *t* test.

The health span of a podocyte can be assessed by changes to its molecular, cellular, and transcriptional landscape required for its normal physiology, structure, and function. To better understand how PD-1 signaling contributes to podocyte aging and how PD-1 might impact podocyte health span, we initially focused on canonical genes essential for the highly specialized structure and function of podocytes. We have previously reported that several of these genes were decreased with aging (26). This was confirmed with the current study cohort. More importantly, the expression of many canonical podocyte genes (e.g., *Actn4*, *Cdkn1*, *Col4a*, *Fat1*, *Lamb2*, *Nphs1*, *Nphs2*, *Podxl*, and *Synpo*) was reduced in aged IgG2a-injected mice, but significantly upregulated upon injection with aPD1ab (Figure 8A). This was confirmed by immunostaining for the podocyte structural proteins nephrin (*Nphs1*) and synaptopodin (*Synpo*) (Figure 8, B–I).

We also analyzed VEGFA as a surrogate for podocyte synthetic function, which is critical for maintenance of podocyte/endothelial cell interaction (41). *Vegfa* mRNA levels were 4.2-fold lower in aged podocytes than in young podocytes, but were 3.4-fold higher in podocytes from aged aPD1ab-injected mice (Figure 8A). The decreased *Vegfa* mRNA expression was validated at the protein level by immunostaining (Figure 8, J–M). The same was observed for another pathway critical for podocyte function, tight junction formation. Again, KEGG pathway analysis revealed that many of the genes involved in tight junction formation were restored in the podocytes of aged mice injected with aPD1ab compared with IgG2a-injected controls (Supplemental Figure 4).

Finally, we investigated the transcription factor drivers of these expression changes in podocytes. Compared with control aged IgG2a-injected mice, several podocyte transcription factors were expressed at significantly higher levels in aged mice injected with aPD1ab. These included *Lmx1b* (4.3-fold), *Osr2* (7-fold), and *Foxc2* (2-fold). The restoration of the podocyte gene regulatory network was further underscored by a VIPER (virtual inference of protein activity by enriched regulon) analysis, which identifies transcription factor activities based on the expression of their downstream targets (26, 42). As shown in Figure 8N, of the top 10 active transcription factors identified by VIPER (sorted by permutation *P* value), the transcriptional activity of 4 (*Hnf1b*, *Nr2f6*, *Mbd3*, and *Tada2b*) was increased while 6 were decreased (*Zfp39*, *Sall1*, *Ets1*, *Tcf4*, *Sox7*, *Zeb2*) upon aPD1ab injection.

Taken together, these data show that the expression levels of canonical podocyte genes and the function and transcriptional regulation required for their health span are partially restored with aPD1ab.

### PD-1 inhibition reduces apoptosis, pyroptosis, and endoplasmic reticulum stress and increases autophagy.

**Figure 9:**
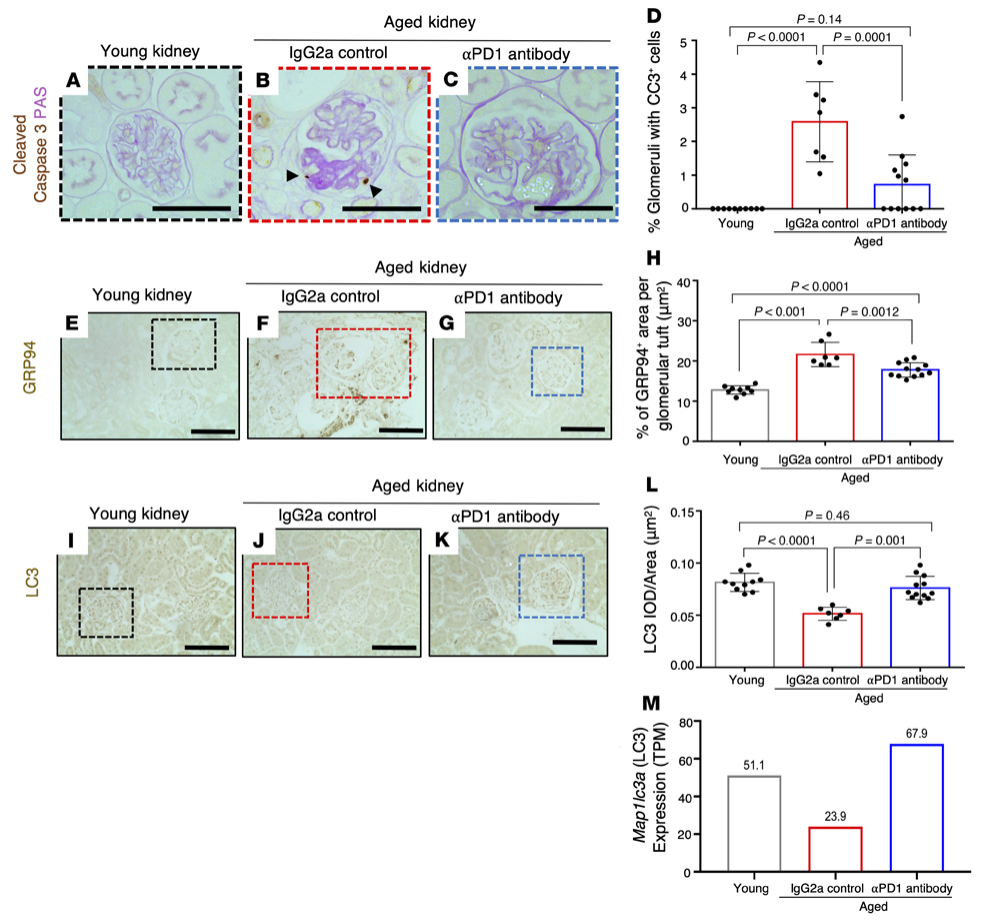
Apoptosis, ER stress, and autophagy. (**A**–**D**) Representative images of immunoperoxidase staining for the apoptosis marker cleaved caspase-3 (brown) and periodic acid counterstain (pink). Glomerular apoptotic cells in aged IgG2a-injected mice are indicated by black arrowheads (**B**). Quantification of the percentage of glomeruli with at least a single cleaved caspase-3–positive (CC3^+^) cell (**D**) shows an increase in aged IgG2a-injected mice (red bar) compared with young mice (gray bar) and a decrease with aPD1ab treatment (blue bar). (**E**–**H**) Representative images of immunoperoxidase staining for the ER stress marker GRP94 (brown) and quantification thereof. GRP94 shows higher staining in aged IgG2a-injected mice (red bar), which was lowered upon aPD1ab treatment (blue bar). Individual mice are represented by circles. (**I**–**M**) Measurement of autophagic activity measured by LC3. Representative images of immunoperoxidase staining for microtubule-associated protein 1 light chain 3 (LC3) (**I**–**K**, brown) and quantification of the staining (**L**). Compared with young mice (gray bar), LC3 staining was lower in IgG2a-injected mice (red bar), indicating reduced autophagy, but was higher with aPD1ab treatment (blue bar). IOD, integrated optical density. (**M**) mRNA-Seq data from isolated podocytes showed a decrease in the *Map1lc3a* (LC3) transcript in IgG2a-injected mice (red bar) compared with young mice (gray bar) but an increase with aPD1ab treatment (blue bar). Scale bars: 50 μm. Statistical analysis was performed by *t* test.

The ectopic expression of PD-1 in podocytes in vitro induced high levels of apoptosis that were in part reduced by inhibition of caspase-3 (Figure 2). Moreover, reduced podocyte number in aging (and disease) is due to increased cell death in the face of an inability to self-renew (43). Indeed, activated caspase-3 staining in podocytes was increased in aged IgG2a-injected control mice (2.6 ± 1.2, *P <* 0.0001 vs. control) and reduced upon aPD1ab injection (0.7 ± 0.9, *P =* 0.0001) (Figure 9, A–D). This was confirmed by mRNA-Seq data, where transcripts for apoptosis genes were elevated (e.g., *Tp53*, 2-fold; *Tnf*, 1.35-fold; *Bim1*, 1.9-fold; *Card10*, 2.5-fold; and *Card14*, 1.8-fold) in aged control mice and reduced in the aPD1ab-injected group. Interestingly, we also observed changes in pyroptosis, another form of podocyte death (44).

Besides cell death, we looked at other forms of cellular stress,such as endoplasmic reticulum stress (ERS) and autophagy (45, 46). The staining intensity of the ERS-associated protein GRP94/Hsp90b1 increased in control IgG2a aged podocytes and was reduced in aged mice injected with aPD1ab (Figure 9, E–H). Conversely, the autophagy protein ATG8/MAP1LC3 (microtubule-associated protein 1 light chain 3 [LC3]), a marker of autophagic activity, showed lower staining in glomeruli of control IgG2a aged mice (*P <* 0.0001 vs. young) and was completely restored in aPD1ab-injected aged mice (*P =* 0.001 vs. aged control) (Figure 9, I–M). Together, these data suggest that anti–PD-1 treatment of aged podocytes results in improved survival, reduced ER stress, and augmented autophagy.

### Age-regulated podocyte signaling can be restored by anti–PD-1 treatment.

We have recently reported that the podocyte aging phenotype is caused by changes in autocrine and paracrine signaling molecules (26). Thus, we wondered whether anti–PD-1 injection altered this signaling network. Aged podocytes exhibit a striking inflammatory transcriptomic signature (26). KEGG pathway analysis (using an FDR cutoff of 0.05) showed that compared with age-matched IgG2a-injected controls, aged mice injected with aPD1ab exhibited a marked decrease in multiple inflammatory pathways (e.g., Toll-like receptor [TLR], IFN-α and -γ, inflammatory genes, complement, allograft rejection, IL-6/JAK/STAT, IL-2/STAT, and KRAS) (Supplemental Figure 5A). This was confirmed at the protein level by immunostaining for TLR4 (Supplemental Figure 5, B–D). Similarly, 9 of the previously reported 52 ligand-receptor pairs regulated in aged podocytes (26) were impacted by aPD1ab injection (Supplemental Figure 6).

### Anti–PD-1 antibody improves the podocyte’s metabolic state and reduces intracellular inflammation.

Next, we performed a gene set enrichment analysis of the mRNA-Seq data to obtain a deeper understanding of the molecular pathways altered upon aPD1ab treatment. The podocytes from mice injected with aPD1ab displayed decreases in T cell activation, positive regulation of protein kinase activity, mitotic nuclear division, DNA repair, and calcium ion transport and marked increases in many metabolic pathways, such as oxidative phosphorylation, amino acid metabolism, organic acid transport, mitochondrial translation, and glucose metabolism (Supplemental Figure 7, A and B). The latter was confirmed by Hallmark pathway analysis, which, in addition to many metabolic pathways (e.g., oxidative phosphorylation, fatty acid metabolism, glycolysis, peroxisome), also identified regulation by the E2F transcription factors as Gene Ontology terms upregulated in aged mice injected with aPD1ab compared with IgG2a (Supplemental Data Sets 1–4). Most impressively, overlaying those data onto individual pathways showed that aPD1ab injections caused upregulation of many key components of oxidative phosphorylation, glycolysis/gluconeogenesis, and the TCA cycle (Supplemental Figure 7C and Supplemental Figures 8 and 9). Together these analyses suggest that one of the primary effects of interfering with PD-1 signaling is a restoration of the metabolic profile of podocytes.

### Immune checkpoint inhibitors do not alter podocyte histology in human kidneys.

PD-1 inhibitors are part of the wider group of immune checkpoint inhibitors (ICPIs), and their use is connected with the occurrence of acute kidney injury (AKI) and, in particular, tubulointerstitial nephritis (47, 48). While morphologic damage to the podocyte as assessed by podocyte foot process effacement and/or proteinuria is clinically not common, we wanted to evaluate this empirically. To this end, we investigated the clinicopathologic features of ICPI-AKI kidney biopsies within our health system. We queried the electronic health record to identify the 10 most recent consecutive cases of patients with kidney biopsy and ICPI treatment. As shown in Supplemental Table 1, almost all these patients presented with elevated serum creatinine and AKI, and only 1 patient presented with proteinuria. None of these patients demonstrated substantial immune complex deposition within their glomeruli by immunofluorescence microscopy. Electron microscopy evaluation revealed that most of these patients had generally preserved podocyte foot processes, with only 1 demonstrating segmental foot process effacement. Unfortunately, while podocyte foot process effacement is widely accepted to indicate podocyte injury, there are no accepted histologic features that would indicate improved podocyte health in response to ICPI therapy on a kidney biopsy.

### Extraglomerular effects of anti–PD-1 signaling on aging.

Interference with PD-1 signaling in mice is a systemic treatment, and thus its effects on cellular aging would not be restricted to the glomerulus. Indeed, tubular epithelial cell senescence assessed by SA-β-gal staining and immunostaining for the senescent proteins p16 and p19 was lowered upon aPD1ab injections (Figure 3). In line with these observations, the mRNA-Seq analysis of the non-podocyte fraction (i.e., the dissociated kidney cells remaining after MACS isolation of podocytes) showed upregulation of the Gene Ontology terms amino acid metabolism, lipid catabolic process, oxidative phosphorylation, and mitochondrial translation in the aPD1ab-injected kidneys, as well as downregulation of processes such as epithelium morphogenesis, regulation of cell development, calcium ion transport, RAS protein signal transduction, and leukocyte differentiation (data not shown). Similarly, Hallmark pathway analysis showed decreases in epithelial-mesenchymal transition, TGF-β signaling, several inflammatory pathways (e.g., TNF-α, NF-κB, IL-2/STAT5, complement signaling, IL-6/JAK/STAT3), and hypoxia. As in the podocytes (Supplemental Figure 7C and Supplemental Figures 8 and 9), many metabolic pathways were higher in aged mice injected with aPD1ab (data not shown). Immunostaining for collagen IV and IL-17A demonstrated that these changes were present in both the interstitium and the kidney epithelial cells, respectively (Supplemental Figure 10).

**Figure 10:**
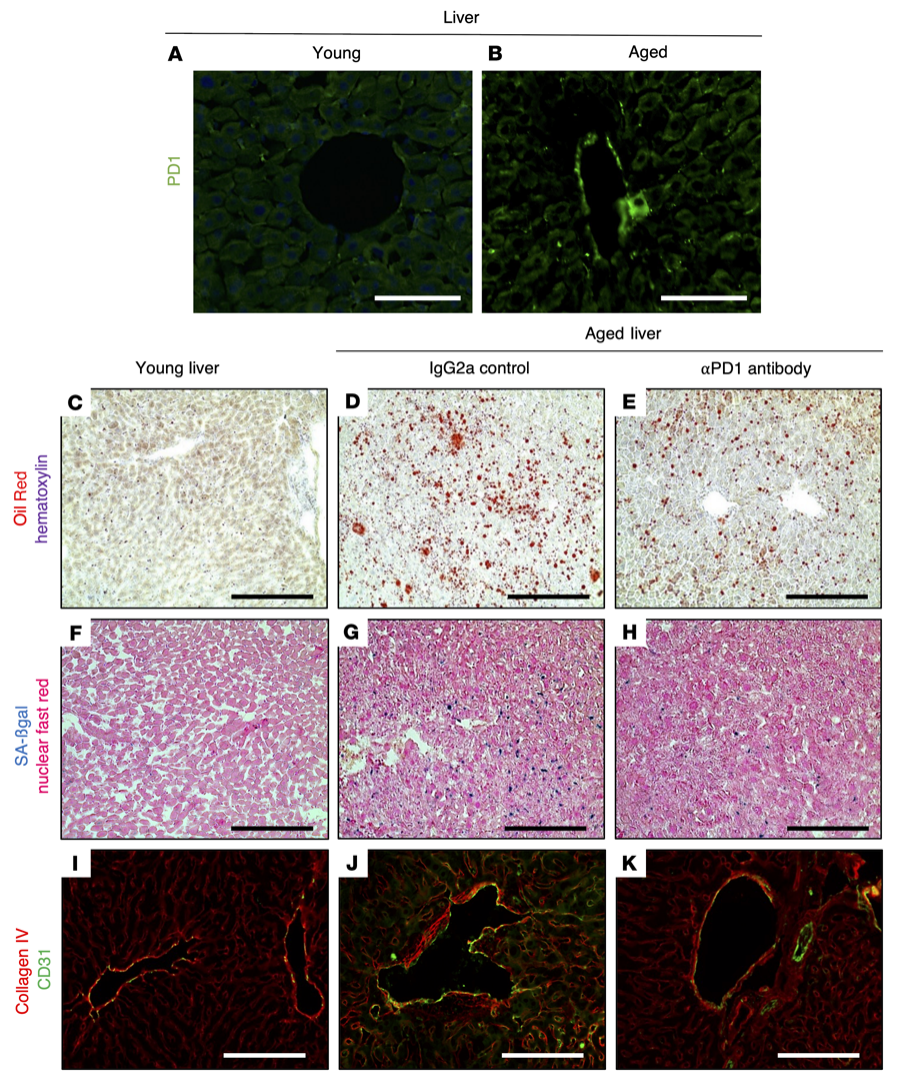
Anti–PD-1 antibody decreases liver aging in mice. (**A** and **B**) Representative images of PD-1 immunofluorescence staining, which is higher in aged mouse liver (**B**) compared with young liver (**A**). (**C**–**E**) Oil Red staining (red) as a marker of fat deposition was barely detected in young livers but was increased in the livers of aged IgG2a-injected mice and decreased in aPD1ab-injected mice. (**F**–**H**) SA-β-gal staining (blue) used as a marker of senescence was increased in aged IgG2a-injected livers and decreased by aPD1ab treatment. (**I**–**K**) Double immunostaining for collagen IV (red) and the endothelial cell marker CD31 (green) shows increased collagen IV deposition in the blood vessels of the liver from aged IgG2a-injected mice compared with young mice, which was decreased by aPD1ab injection. Scale bars represent 50 μm (**A** and **B**) and 100 μm (**C**–**K**).

Finally, by extending our analysis to the liver, we observed a similar anti-aging effect outside of the kidney. PD-1 protein was present in the liver of aged mice, but not young mice (Figure 10, A and B). Moreover, aPD1ab injection reverted the age-associated increase in liver fat deposition (Oil Red staining), senescence (SA-β-gal staining), and extracellular matrix deposition (collagen IV immunostaining) in comparison with control IgG2a-injected mice (Figure 10, C–K). Together, these data suggest that inhibition of PD-1 signaling was not restricted to the kidney and that aPD1ab injections have a more widespread benefit on aging phenotypes.

### Anti–PD-1 antibody improves outcomes in experimental focal segmental glomerulosclerosis.

**Figure 11:**
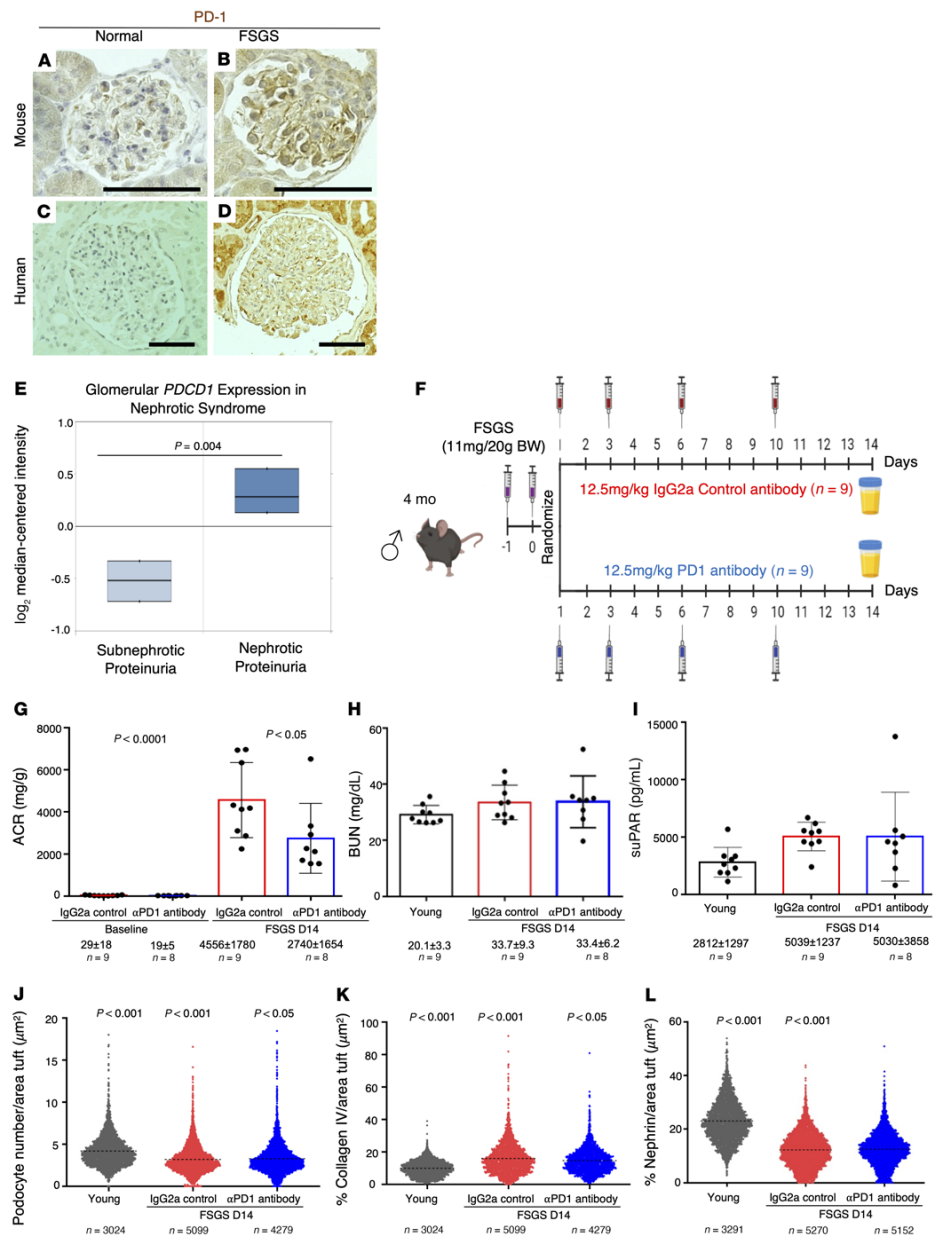
Anti–PD-1 antibody improves experimental FSGS. (**A**–**D**) PD-1 immunoperoxidase staining (brown) of glomeruli from normal young mice (**A**) is increased in young mice with experimental FSGS (**B**). Similarly, in humans, PD-1 staining of glomeruli of a young human kidney (**C**) is increased in podocytes, PECs, and tubular epithelial cells of a kidney from an FSGS patient (**D**). Scale bars represent 50 μm. (**E**) Analysis of the Nephroseq data from NEPTUNE shows that *PDCD1* mRNA levels in human microdissected glomeruli are significantly higher in patients with nephrotic-range versus sub-nephrotic-range proteinuria (*n =* 38). (**F**) To study PD-1 signaling in FSGS, 4-month-old mice were injected twice i.p. with a sheep anti-glomerular antibody. Mice were randomized into 2 groups, which received either an anti–PD-1 antibody (*n =* 8) or the isotype control IgG2a (*n =* 7) on days 1, 3, 6, and 10 after FSGS induction. (**G**–**I**) At the conclusion of the experiment, kidney function analyses showed that albumin/creatinine ratio (ACR) values significantly increased following FSGS induction in IgG2a control–injected but were reduced in aPD1ab-injected mice (**G**). Blood urea nitrogen (BUN) levels were not significantly different between the groups (**H**), while plasma soluble urokinase plasminogen activator receptor (suPAR) levels were significantly increased in FSGS mice injected with control IgG2a compared with young mice but were not reduced in mice injected with aPD1ab (**I**). (**J**) Quantification of podocyte density was lower in FSGS mice injected with IgG2a compared with young mice and was increased upon aPD1ab injection. (**K**) Glomerular scarring measured by glomerular collagen IV staining was higher in FSGS mice injected with IgG2a compared with young mice and was lowered by aPD1ab injection. (**L**) Nephrin immunostaining was lower in FSGS mice injected with IgG2a compared with young mice but was not significantly changed by aPD1ab injection. Each circle in **J**–**L** represents an individual glomerulus, and the number of glomeruli quantified is indicated. Statistical analysis was performed by *t* test.

Aging and glomerular disease share some common senescence features (49). Thus, we wondered whether PD-1 signaling also had a role in glomerular disease of non-aged kidneys. To this end, we induced experimental focal segmental glomerulosclerosis (FSGS) in young mice and measured PD-1 expression. Indeed, glomerular immunostaining for PD-1 was much higher in the glomeruli of experimental FSGS mice compared with control (Figure 11, A and B). The same was observed in samples of human FSGS (Figure 11, C and D). Furthermore, we searched the Nephroseq database for *PDCD1* mRNA expression in human microdissected glomeruli from patients undergoing indication kidney biopsies enrolled in the Nephrotic Syndrome Study Network (NEPTUNE). *PDCD1* expression was significantly higher in patients with nephrotic-range proteinuria versus sub-nephrotic-range proteinuria (50) (*P* = 0.004, *n =* 38 samples; Figure 11E).

We next administered the same anti–PD-1 antibody used in the aging studies to mice with experimental FSGS (Figure 11F). When glomerular function was measured at day 14, FSGS mice treated with the control antibody had proteinuria in the nephrotic range, which was significantly lowered by the anti–PD-1 treatment (Figure 11G). Blood urea nitrogen did not increase in this model (Figure 11H). In line with other experimental models of FSGS and clinical FSGS (51–53), serum soluble urokinase plasminogen activator receptor (suPAR) levels increased in mice with experimental FSGS. However, suPAR levels were unchanged in FSGS mice treated with aPD1ab (Figure 11I). Podocyte number (Figure 11J) was higher and glomerular collagen IV staining (Figure 11K) was lower in FSGS mice receiving aPD1ab, with no change in nephrin staining (Figure 11L). Together, these results suggest that anti–PD-1 treatment not only antagonizes aging but also improves outcomes in young mice with experimental FSGS.

## Discussion

Many tumors evade being detected by suppressing T cell immune responses upon activation of negative regulatory pathways called immune checkpoints. The latter include programmed cell death protein 1 (PD-1) and its ligands, PD-L1 and PD-L2. The development of checkpoint inhibitors for the PD-1 pathway restores T cell recognition and killing of cancer cells (30, 31, 54). However, much less is known about the PD-1 signaling pathway in non-tumor cells. Building on our recent report that PD-1 and its ligands are increased in aged mouse podocytes (26), we provide human data that glomerular *PDCD1* transcripts increase progressively with human aging, and that this increase is statistically correlated with lower eGFR, higher segmental glomerulosclerosis, and vascular damage. The current study asks whether the glomerular consequences of increased PD-1 can be slowed or even reversed in the aged mouse kidney. We show that blocking PD-1 with an antibody improves the aging phenotype in kidneys and liver in mice, with a particular improvement in both the life span and health span of aged podocytes. This interpretation is based on 5 major findings: (a) Aged mouse and human kidneys displayed higher levels of PD-1 immunostaining in epithelial cells (podocytes, PECs, tubular cells), but not in glomerular mesangial and endothelial cells. (b) Ectopic expression of PD-1 in cultured podocytes triggered an apoptotic response that was partially caspase-3 dependent. (c) Interfering with PD-1 signaling in mice using a neutralizing anti–PD-1 antibody reduced senescence markers in the kidney glomerulus, tubular epithelial cells, and the tubulointerstitium as well as the liver. (d) While interfering with PD-1 signaling reversed some aspects of aging, it did not reverse all effects. (e) Finally, blocking PD-1 signaling improved podocyte life span in an experimental model of FSGS.

One interesting aspect of the study is interpretation of the data with respect to the health span and life span of podocytes, which are critical to the function and number of aging podocytes (25). Injecting aPD1ab into aged mice improves the life span (i.e., survival) of podocytes by decreasing several proapoptotic and pyroptosis genes and increasing survival genes. For example, increased cleaved caspase-3 was reduced in podocytes of mice treated with aPD1ab. Moreover, our cell culture studies support the notion that caspase-3 is a pathway underlying PD-1–induced apoptosis. Yet the precise contributions of other processes that reduce podocyte life span (alternate apoptosis pathways, pyroptosis, detachment, and mitotic catastrophe) by PD-1 signaling still need to be resolved (25). In addition, the mouse studies demonstrate that PD-1 inhibition restores many of the features downregulated in aging podocytes required for their normal function (health span). These include not only individual canonical podocyte genes (e.g., *Nphs1*, *Nphs2*, *Synpo*, and *Vegfa*) or signaling networks (e.g., FGF signaling), but entire podocyte gene regulatory networks as illustrated by the VIPER analysis. Likewise, PD-1 inhibition improved the senescent and inflammatory phenotypes present in aged podocytes, which improved podocyte health span. Restoring these probably has a long-lasting effect above simply preventing podocyte death. In this regard, the observation that aPD1ab injections restored normal metabolic pathways such as oxidative phosphorylation, glycolysis, and lipid metabolism further supports the pro–health span effects of PD-1 signaling inhibition. There may also be other consequences that improve the health of podocytes. For example, we have recently reported that aging podocytes exhibit not only autocrine loops, but also paracrine loops, in which podocytes signal to other cell types in the glomerulus (26). In the current study we observed that interfering with PD-1 signaling did not impact the age-dependent changes in glomerular mesangial or endothelial cell numbers, but injection of aPD1ab reduced the activated PEC phenotype (decreased CD44, CD74, and p-ERK) and the stressed GEN phenotype (decreased PV-1). It is thus tempting to speculate that this “restoration” occurs via paracrine loops emanating from podocytes restored by the aPD1ab treatment. Indeed, our data on VEGFA as a surrogate marker for podocyte-endothelial crosstalk support this hypothesis. However, given the pronounced cell death phenotype observed following an increase in PD-1 signaling in cultured podocytes (Figure 2), more wide-ranging experimental analysis of compound mutants will be needed to address the individual contributions of these pathways in the future.

Studies have shown an association between increased PD-1 pathway expression and aging. The PD-1/PD-L1 pathway has been shown to be increased in aged dendritic cell subtypes and T cells (55, 56) and CD4^+^ T cells with features of cellular senescence (57) and increases further with age in many cancers (58). The PD-1/PD-L1 pathway is also increased in several kidney diseases independent of age, but expression is typically restricted to immune cells (reviewed in ref. 59). Kidney interstitial dendritic cells and human primary kidney proximal tubular epithelial cells express PD-L1 and PD-L2, where PD-L1 expression is integral for CD8^+^ T cell tolerance (60). Moreover, for the first time to our knowledge, we show that increased expression of *PDCD1* in aging human glomeruli is highly clinically relevant, correlating with lower kidney function, higher scarring, and increased vascular damage.

PD-1 immunostaining increased in glomeruli of young mice and young patients with FSGS, in a predominantly podocyte distribution. Administering the same anti–PD-1 antibody used in the aging studies to young mice with experimental FSGS lowered proteinuria and glomerular collagen IV staining, accompanied by a higher podocyte density. Similarly to experimental and clinical FSGS (51–53), plasma suPAR increased in this model of experimental FSGS, but was not changed by the anti–PD-1 antibody.

Hydrogel expansion with FLARE staining (61) and PEMP (35) provided ultrastructural analysis of the filtration barrier and podocytes. We observed dramatic thickening of the glomerular basement membrane and a decrease in filtration slit density with aging. While there was some improvement with anti–PD-1 treatment, neither was completely restored to the level seen in young animals. A few possible explanations include: (a) as these changes were likely well established prior to the relatively short (8 weeks) anti–PD-1 treatment period, there may not have been sufficient time to reverse the changes; (b) improvements in podocyte health span measurements do not reflect a complete reversal of the ultrastructural damage; (c) if aPD1ab treatment primarily improves the podocyte life span, a decrease in podocyte loss would not automatically reflect a reversal of the ultrastructural damage of the remaining podocytes.

The study and usage of senolytics, i.e., drugs that selectively clear senescent cells from an aging patient/organ, is a vibrant field (62). For example, the cancer drugs quercetin and dasatinib, which are broad-spectrum inhibitors of protein kinases and tyrosine kinases, have been shown to reduce markers of aging (63–65). Indeed, dasatinib and quercetin improved kidney function, increased expression of WT1, and decreased p16 levels in a diabetic model of senescence (66). Similarly, induction of senescence-associated secretory phenotypes (SASPs) in aging cells has been a much-discussed anti-aging target (67). Yet the heterogeneity of SASP cytokines and differences in SASPs from cell type to cell type have made it difficult to develop viable anti-aging therapies. Thus, the focus has been toward some shared intracellular targets. Our study now suggests that there may be an alternative avenue. Interfering with PD-1 in aged kidneys reduces the SASP of aging podocytes by markedly decreasing inflammatory pathways. Thus, upstream regulation of SASP may be a future avenue for anti-aging therapies.

We recognize that widespread administration of immune checkpoint inhibitors is unlikely to be a generalized anti-aging strategy. These agents can cause renal toxicity (68–70), with an estimated incidence of acute kidney injury of 2%, typically due to immune overactivation resulting in tubulointerstitial nephritis (71). Still, it is noteworthy that in the current study, anti–PD-1 antibody administration in aged mice reduced KIM-1 protein, a well-established marker for tubular injury. Similarly, in silico analysis of the transcriptomics data of the non-podocyte fraction, which comprised a large number of proximal tubular cells, showed reduced gene expression of several tubular injury–related genes (e.g., *Havcr1*, *Lcn1*, and *Vcam1*). These data are consistent with the interpretation that the anti–PD-1 antibody improves rather than harms the tubulointerstitium. Similarly, with respect to the glomerulus, individual case reports of glomerulopathies as a complication of anti–PD-1 immunotherapy exist (60, 72), though podocyte injury as assessed by foot process effacement was not common in our kidney biopsy cohort. We cannot exclude the possibility of stabilization or improvement of podocyte phenotypes in our patient cohort, since histologic features of improved podocyte health are not well accepted.

We recognize the limitations of the current study, which include that anti–PD-1 antibody treatment was given systemically rather than in a cell-specific targeted manner and was given only for a short duration. Yet the impact on the kidney was impressive for both aging and FSGS. However, the impact on organs beyond the kidney and liver was not assessed in this study. Finally, one puzzling feature of our study is that the very small amount of microalbuminuria, normally present in mice, was not statistically elevated in aged mice compared with young mice; similarly, other measures of kidney function (blood urea nitrogen and plasma suPAR) were not changed upon aging either. This is in agreement with several reports showing that the majority of mouse strains do not show appreciable microalbuminuria, nor do they develop proteinuria as they age (73–75). In fact, in non-aged, inbred mouse strains, the albumin/creatinine ratio typically ranges from non-detectable to 200 mg/g (74). Importantly, this is consistent with clinical studies in humans. Rule et al. (76) and others (77, 78) have reported that proteinuria is not a feature of aging. Instead, elderly individuals with proteinuria normally exhibit secondary disease manifestations such as hypertension, obesity, or diabetes. There are several possible explanations for the discrepancy between the age-dependent changes in podocytes and their lack of albuminuria: (a) As nephrons are lost with advancing age, the remaining nephrons hypertrophy (79) and thus become more effective in reclaiming filtered albumin (and other nutrients) (80). (b) Following podocyte loss, the remaining podocytes in that glomerulus hypertrophy as much as 10-fold to cover the denuded filtration surface with foot processes (18, 22), which at least initially maintains glomerular filtration characteristics (22). (c) Once podocyte density in individual glomeruli reaches critical levels (i.e., less than 35–50 podocytes per μm^3^ × 10^6^ tuft volume), these glomeruli undergo rapid global glomerulosclerosis due to massive podocyte catastrophe (22, 81). Yet this does not result in detectable albuminuria until a large number of glomeruli are lost very late in life. (d) Studies in rats show an age-dependent increase in the expression of the protein reuptake receptors megalin and cubilin in podocytes and PECs, which may reduce urinary albuminuria with age (82).

In summary, the present study shows that the PD-1 pathway is increased in aged mouse podocytes and in aged human glomeruli, in podocytes of young mice and humans with FSGS, and in aged mouse livers. Increased expression of the PD-1 signaling pathway in human glomeruli predicts poor human kidney outcomes. Mechanistically, increased PD-1 levels in podocytes cause apoptotic loss (shortened podocyte life span) and reduce their health span. These events and glomerulosclerosis are partially reversed by inhibition of PD-1. These results are consistent with PD-1 being a critical mechanism contributing to glomerular damage in the aged kidney and following kidney injury.

## Methods

The methods are described in detail in Supplemental Methods. Animal studies were reviewed and approved by the University of Washington IACUC (2968-04). Human studies were approved by the IRBs of the University of Michigan (HUM00052918) and the University of Chicago (IRB14-0167). All patient samples were deidentified. Clinicopathologic evaluation of kidney biopsies from patients treated with immune checkpoint inhibitors was conducted under University of Washington IRB protocol 2837. Raw and processed mRNA-Seq data were deposited in the NCBI’s Gene Expression Omnibus database (GSE186534) and can be browsed at https://yuliangwang.shinyapps.io/podocyte_PD1_experiment/

## Author contributions

JWP, DGE, MB, OW, JCV, and SJS designed research studies. JWP, NK, YW, DGE, YZ, UT, CJL, CO, CP, HSP, and SA conducted experiments. JWP, NK, YW, DGE, YZ, UT, CJL, CO, CP, HSP, JCV, and SA acquired data. JWP, NK, YW, DGE, CO, MB, OW, SJS, CP, HSP, JCV, and SA analyzed data. JWP, NK, DGE, CO, MB, OW, and SJS wrote the manuscript. AC and SA provided material. SA performed clinicopathologic evaluation of human kidney biopsies.

## Supplementary Material

Supplemental data

Supplemental data set 1

Supplemental data set 2

Supplemental data set 3

Supplemental data set 4

Supplemental video 1

Supplemental video 2

Supplemental video 3

## Version 1 08/15/2022

Electronic publication
