# Supplementary material for: Upregulated PD-1 signaling antagonizes glomerular health in aged kidneys and disease
Source: J Clin Invest. 2022 Aug 15;132(16):e156250. doi: 10.1172/JCI156250 (PMC9374384; doi:10.1172/JCI156250)
Supplement: Supplemental data [file jci-132-156250-s044.pdf]

SUPPLEMENTAL METHODS:

Administering an anti-PD1 Antibody to Aged Mice

21 month-old wildtype male, C57BL/6J mice (Strain #000664, The Jackson Laboratory) (~ 70 year-old humans) (83, 84) were randomized into two groups: (1) anti-PD-1 antibody-injected group (n=12) that received weekly IP injections of InVivoMAb anti-mouse PD-1 antibody (RMP1-14, Bio X Cell, Lebanon, NH) at 12 mg/kg for 8 weeks; (2) aged-matched control mice (n=7) that received weekly IP injections of the isotype control IgG2A antibody (2A3, Bio X Cell, Lebanon, NH) at 12 mg/kg for 8 weeks. A third group of young non injected C57BL/6J mice (Strain #000664, The Jackson Laboratory) aged 4 months (n=9) were used as a non-aged comparison. Animals were weighed weekly; spot urines were collected prior to injections at 4, and 8 weeks; albumin was measured by radial immunodiffusion assay (RID) and urine creatinine was determined with a Creatinine (urinary) Colorimetric Assay Kit (Cayman Chemical, Ann Arbor, MI) (85). Blood Urea Nitrogen was measured at sacrifice, with a colorimetric Urea Assay Kit (Abcam, Cambridge, United Kingdom). Circulating suPAR levels were measured in plasma samples collected at sacrifice, using Mouse uPAR DuoSet ELISA (Cat, #DY531, R&D Systems Minneapolis, MN).

Administering an anti-PD1 Antibody to FSGS Mice

Experimental focal segmental glomerulosclerosis (FSGS) was induced in 4-month-old wildtype male C57BL/6J mice (Strain #000664, The Jackson Laboratory) by two intra-peritoneal (IP) injections, 24 hours apart, of a cytopathic sheep anti-glomerular antibody (11 mg/20 g body weight) (86-88). Animals were immediately randomized into two groups: (1) anti-PD-1 antibody-injected group (n=8) and (2) isotype control IgG2a-injected group (n=7) that received IP injections at 12 mg/kg, on days 1, 3, 6, and 10 post FSGS induction. These were the same antibodies administered to aged mice described above. Urine was collected at baseline and FSGS day 14, blood was collected at sacrifice, and samples were analyzed as described above.

Immunostaining, Quantification and Visualization

Immunoperoxidase staining was performed on 4μm thick formalin fixed paraffin-embedded (FFPE) mouse and human kidney sections as previously described (89). Double immunostaining was performed on 4 μm thick frozen and FFPE sections as previously described (90). These immunohistochemical studies were approved by the University of Chicago institutional review board. The primary antibodies used in the study are summarized in **Supplemental Table 2**. To quantify immunohistochemistry, slides were scanned in brightfield with a 20x objective using a NanoZoomer Digital Pathology System (Hamamatsu City, Japan). The digital images were imported into Visiopharm software (Hoersholm, Denmark) and its Image Analysis Deep Learning module was trained to detect glomeruli and assess immunohistochemical staining-positivity for e.g., Collagen IV, p57 or ERG. The glomeruli ROIs were processed in batch mode generating per area outputs, cell counts and analyzed from 100% of the tissue sections. In the case of the parietal epithelial cells, images were collected using an EVOS FL Cell Imaging System (Life Technologies). Bowman's capsule length was measured by using ImageJ 1.46r software (National Institutes of Health) and the percentage of positivity were calculated by dividing it by Bowman's capsule length.

Two–dimensional images were detected on EVOS FL Cell imaging system (Thermo Fisher Scientific, Waltham, MA, USA) using a 20x objective and increased to a 200x magnification. Stained kidney sections were digitally imaged by the University of Washington Histology and Imaging Core (HIC) using a Hamamatsu whole slide scanner (Bridgewater, NJ, USA).

In situ Hybridization

RNA in situ hybridization was performed on the kidney tissues by following the manufacturer instructions for the RNAscope 2.0 FFPE Assay kit –BROWN (Advanced Cell Diagnostics). Briefly, kidneys were perfused and fixed in 10% formalin, dehydrated and embedded in paraffin. 4 μm paraffin sections were cut, dehydrated in 100% and 95% of Ethanol solutions. Sections were boiled at 100^0C in

EZprep buffer for 20 min followed by a protease incubation for 30 min at 37^{0}C. Probes specific to mouse PDCD1 (Advanced Cell Diagnostics) were hybridized at 48^{0}C for 2 hours in an oven, followed by a subsequent series of washing and signal amplification steps. Mouse specific positive and negative probes for PDCD1 gene were provided by Advanced Cell Diagnostics. Hybridization signals were detected by DAB staining. Stained tissues were imaged on an EVOS FL Cell Imaging System.

FLARE Staining and Expansion Microscopy

Hydrogel-expansion and FLARE staining of kidney sections were performed according to the published FLARE protocol (61) on 50 µm thick sections of cryo-preserved mouse kidney tissue. In brief, oxidized carbohydrates were labeled with ATTO 565 hydrazide, amines (proteins) were labeled with ATTO 647N NHS ester, and nuclei were labeled with the fluorescent DNA-binding dye SYBR Green I. Expanded gels were transferred onto a poly-L lysine coated coverslips (24 mm by 50 mm, no. 1.5; Fisher Scientific, #12544E) and imaged immediately. Images were acquired using a Nikon A1R inverted point-scanning confocal microscope at the University of Washington Biology Imaging Facility. A CFI Apo LWD Lambda S 40× objective lens with 1.15 numerical aperture was used on the microscope. Three-color 3D stacks were acquired with 100 nm lateral sampling and 200 nm axial sampling. A median-filter of 1 pixel radius was applied to all representative images. The glomerular basement membrane thickness was measured using Gaussian fits to line-profiles drawn perpendicularly across the membrane, n=50 measurements per condition. The full width at half-maximum is reported in Figure 3L.

Measuring Filtration Slit Density

After deparaffinization and rehydration, kidney sections (2 µm) were boiled in Tris-EDTA buffer (10 mmol/l Tris, 1 mmol/l EDTA, pH 9) in a pressure cooker for 5 min, followed by a blocking step (1% FBS, 1% BSA, 0.1% fish gelatin, 1% normal goat serum) for 1 hour. The following primary antibodies were incubated overnight at 4°C: rabbit anti-Podocin 1:500 (IBL International), mouse anti-Integrin 3 alpha 1:500 (Santa Cruz Biotechnology). After three washing steps in PBS, secondary antibodies were incubated for 1 hour at room temperature (anti-rabbit Alexa Fluor 488-conjugated IgG 1:600 (ChromoTek) and anti-

mouse Cy3-conjugated IgG antibody 1:600 (Jackson ImmunoResearch) for 1 hours at room temperature. DAPI (1:100) was added to the slides for 5 min, followed by a washing step in PBS. Finally, the slides were incubated in H_2O and mounted in Mowiol (Carl Roth) using high-precision coverslips (Paul Marienfeld). For 3-D SIM, z-stacks of 19 planes of both channels (488 and 561 nm) were acquired from the stained kidney sections using the N-SIM super-resolution microscope (Nikon) equipped with a 100x silicone objective. The images were reconstructed into 3-D SIM images using NIS-Elements AR 5.30 (Nikon). The z-stacks were converted into a maximum intensity projection (MIP) followed by the automatized identification of the filtration slit length. The filtration slit density (FSD), meaning the length of the filtration slit per podocyte foot process area, was determined. This podocyte exact morphology measurement procedure (PEMP) has been described in detail previously (35). The FSD of 20 glomeruli was determined (n=5/group).

All graphs were set up using Prism 9 (GraphPad Software). Normality of the groups was tested using Shapiro-Wilk normality test. The groups were compared using one-way ANOVA corrected for multiple comparison by controlling the false discovery rate (FDR) using the method of Benjamini and Hochberg. FDR-adjusted p-values of less than 0.05 were considered significant.

Assessment of Liver Aging

Fresh liver biopsies were preserved by snap freezing in OCT and stored at -80 C. Frozen sections were cut (8 µm thick) and used for staining as prescribed above. Hepatic lipids and triglycerides were determined by Oil Red O staining as previously described (91). To determine morphological changes present in aged liver, frozen liver sections (6µm) were stained for Collagen IV and the endothelial marker CD31/PECAM-1 as previously described (92).

Magnetic Activated Cell Sorting (MACS) of Podocyte and Non-Podocyte Cell Fractions

Kidney tissue (w/o the kidney capsule and surrounding fat) was placed into ice cold RPMI 1640 medium (w/o L-glutamine and phenol red, GE Healthcare Bio-Sciences, Pittsburgh, PA). After removal

of the medulla, the remaining cortex was minced into fine pieces and digested in 0.2 mg/ml Liberase™ TL (Sigma-Aldrich, St. Louis, MO), 100 U/ml DNAse I (Sigma-Aldrich, St. Louis, MO) in RPMI 1640 medium (w/o L-glutamine and phenol red) by shaking at 37°C for 30 minutes. The digest was passed through an 18G needle (Becton Dickenson, Franklin Lakes, NJ) 10 times and enzymes were inactivated by adding 5 ml of RPMI 1640 medium (w/o L-glutamine and phenol red) supplemented with 1 mM sodium pyruvate (ThermoFisher Scientific, Waltham, MA), 9% Nu-Serum™ IV Growth Medium Supplement (Corning Incorporated - Life Sciences, Durham, NC) and 100U/ml Penicillin-Streptomycin (ThermoFisher Scientific, Waltham, MA). The cell suspension was passed through a 100 µm and a 40 µm cell strainer (BD Biosciences, San Jose, CA) and pelleted by centrifugation at 200G at 4°C for 5 minutes. Cells were were resuspended in media containing two rabbit anti-Nephrin antibodies (93) (1:100, Abcam, Cambridge, MA). After 1 hour at 4°C, cells were pelleted, washed in media and incubated with anti-rabbit microbeads (Miltenyi Biotec, Auburn, CA) along with Alexa Fluor® 594-conjugated AffiniPure Donkey Anti-Rabbit IgG 1:200 (in order to visualize binding of the Nephrin antibodies to the podocytes) for 30 minutes at 4°C. Cells were pelleted and washed in PBS with 0.5% BSA and 2mM EDTA and applied to MACS LS columns (Miltenyi Biotec, Auburn, CA) to gently separate microbead-bound podocytes from the other kidney cells. Cells not retained by the magnetic field were collected, pelleted and designated non-podocyte (NP) fractions. LS columns were removed from the magnetic field then washed with PBS with 0.5% BSA and 2 mM EDTA and podocytes were collected. A small aliquot of each fraction were imaged using an EVOS FL Cell Imaging System to verify podocyte isolation, based on the presence of Nephrin antibody. Additionally, qRT-PCR for a panel of podocyte genes was performed in both podocyte and non-podocyte fractions to confirm cell type identity.

RNA isolation, qRT-PCR, Library Preparation and Sequencing

mRNA was isolated using the RNeasy Mini Kit (Qiagen, Germantown, MD) as per the manufacturer's instructions and used for bulk mRNA sequencing or converted to cDNA by reverse transcription with the High-Capacity RNA-to-cDNA Kit (Thermo Fisher, Waltham, MA) and utilized for

quantitative real-time PCR analysis. qRT-PCR was performed using iTaq SYBR Green Supermix (Bio-Rad, Hercules, CA) and a QuantStudio 6 Flex real-time PCR System (Applied Biosystems) as we have previously described (26). Relative mRNA expression levels were normalized to *glyceraldehyde-3-phosphate dehydrogenase* (*Gapdh*) levels. Library generation and bulk next-generation mRNA sequencing was performed by Psomagen, Inc. (Rockville, MD, USA) using TruSeq RNA Library Prep Kits (Illumina, San Diego, CA, USA) and the Illumina platform. Data analysis of the mRNA-seq data was performed as follows:

(1) Raw reads from fastq files were aligned to mm10 using the SubRead aligner (94). Gene-level read counts were obtained using htseq-count (95). Genes with less than 10 normalized reads summed across all samples were removed from further analysis. DESeq (96) was used to identify differentially expressed genes (DEGs), which were defined as genes with false discovery rate <0.05 and >2-fold change comparing young to aged. We additionally required that a DEG should have mean expression above 4 RPKM in either young or aged mice.

(2) Gene Set Enrichment Analysis (GSEA) was used to identify perturbed biological processes (97). Genes were first ordered based on the pi score, a metric that combined p-value and fold change (98). Genes with both a large increase in expression in aged podocytes and high statistical significance were at the top of the list; genes with both a large decrease in expression and high statistical significance were at the bottom of the list. Genes with moderate expression fold change and/or statistical significance were ranked in the middle. This ranked gene list was then used as input for GSEA.

(3) Additionally, the top Gene Ontology (GO) package (99) was used for GO enrichment analysis, based on Fisher's exact test. In order to visualize the interactions between genes within perturbed pathways, we mapped DEGs onto network diagrams from the KEGG pathway database (100) using the PathView tool (101).

(4) The VIPER *(*virtual inference of protein activity by enriched regulon analysis*)* method was used to identify potential master transcriptional regulators of podocyte aging as we described previously (42)

Genes were sorted from the most down-regulated genes in aged podocytes to the most up-regulated genes in aged podocytes compared to young podocytes. If a significant fraction of a TF's positive targets were up-regulated, and its negative targets down-regulated in aged podocytes, this TF is inferred to be activated in aged podocytes (inactivated in young podocytes); if a significant fraction of a TF's positive targets are down-regulated and its negative targets are up-regulated in aged podocytes, the TF is inferred to be inactivated in aged podocytes (activated in young podocytes).

PD-1 Overexpression in Mouse Podocytes

The Lenti-X 293T Cell Line (Takara Bio USA, Inc., Mountain View, CA) was cultured according to the manufacturer's instructions and transfected with 5 µg pLenti-C-mGFP-P2A-Puro (control) or pLenti-C-mGFP-PD1-P2A-Puro (*Pdcd1* Overexpression), 5 µg of pLenti-Envelop vector and 6 µg of packaging plasmids (OriGene Technologies, Inc. Rockville, MD) to isolate lentivirus according to the manufacturer's instructions. Immortalized mouse podocytes were isolated and cultured as previously described in detail (32-34) and infected with either pLenti-GFP control or pLenti-PD1 viral particles for 7 days and selected for expression using GFP expression via FACS. To confirm PD1 overexpression, mRNA was harvested and qPCR performed as described above. To block PD1, InVivoMAb anti-mouse PD-1 antibody (RMP1-14, Bio X Cell, Lebanon, NH) was applied every other day at 10 µg/ml culture media for 5 days. To inhibit Caspase 3, the Caspase 3-specific inhibitor Z-DEVD-FMK (FMK004, R&D Systems, Minneapolis, MN) was applied at 100µm concentration for 72 hours. DMSO vehicle served as control. To measure podocyte death, 40x or 100x random images were captured using an EVOS FL Cell Imaging System (Life Technologies) from 6 replicate samples per experimental group. Utilizing EVOS FL Auto 2 Software**,** the auto count feature was used to identify and count dead cells. Immunocytochemistry was performed on 2% paraformaldehyde 4% sucrose fixed podocytes permeabilized with 0.3% Triton x-100. Rabbit antibodies to msPD1 (50124-RP02, Sino Biologicals, Wayne, PA), PD1L1(64988S Cell Signaling Technologies, Danvers, MA) and cleaved-Caspase 3 (9579S, Cell Signaling Technologies) were applied and visualized with Alexa Fluor® 594 AffiniPure Donkey Anti-Rabbit IgG (Jackson ImmunoResearch, West Grove, PA).

Gene Expression Analysis of Human Kidneys

Kidney tissue was obtained from the unaffected parts of kidneys removed from patients undergoing surgery at the University of Michigan and processed via the tissue procurement service of the Department of Pathology. Clinical data were obtained through the honest broker office of the University of Michigan as we have reported (102, 103). Tissue was placed right away in RNAlater, micro-dissected into glomeruli and tubulo-interstitial fractions, and isolated RNA was used for gene expression analysis using Affymetrix Human Gene 2.1 ST Array (22, 103). This study was approved by the Institutional Review Board of the University of Michigan.

Injury Analysis of Human Kidney Biopsies

Young kidneys consisted of nephrectomy specimens from trauma victims under the age of 30 years, which demonstrated normal renal parenchyma. Aged kidney specimens from patients greater than 70 years of age with tubulointerstitial injuries (acute interstitial nephritis or acute tubular necrosis) were utilized. Focal segmental glomerulosclerosis (FSGS) specimens were generally of the not otherwise specified variant and all patients had nephrotic-range proteinuria. To determine if the clinical usage of immune checkpoint inhibitor therapy (ICPI) affected human podocytes, we queried the electronic health record to identify the ten most recent consecutive cases of patients with kidney biopsy and ICPI treatment at the University of Washington Medical Center. The kidney biopsies were examined closely for podocyte changes.

Statistical Analysis

Data are shown as the mean ± S.E.M. Student's t-test was applied for comparisons between groups. Multiple groups were compared using one-way ANOVA with post hoc Tukey HSD test. P values <0.05 were considered statistically significant differences.

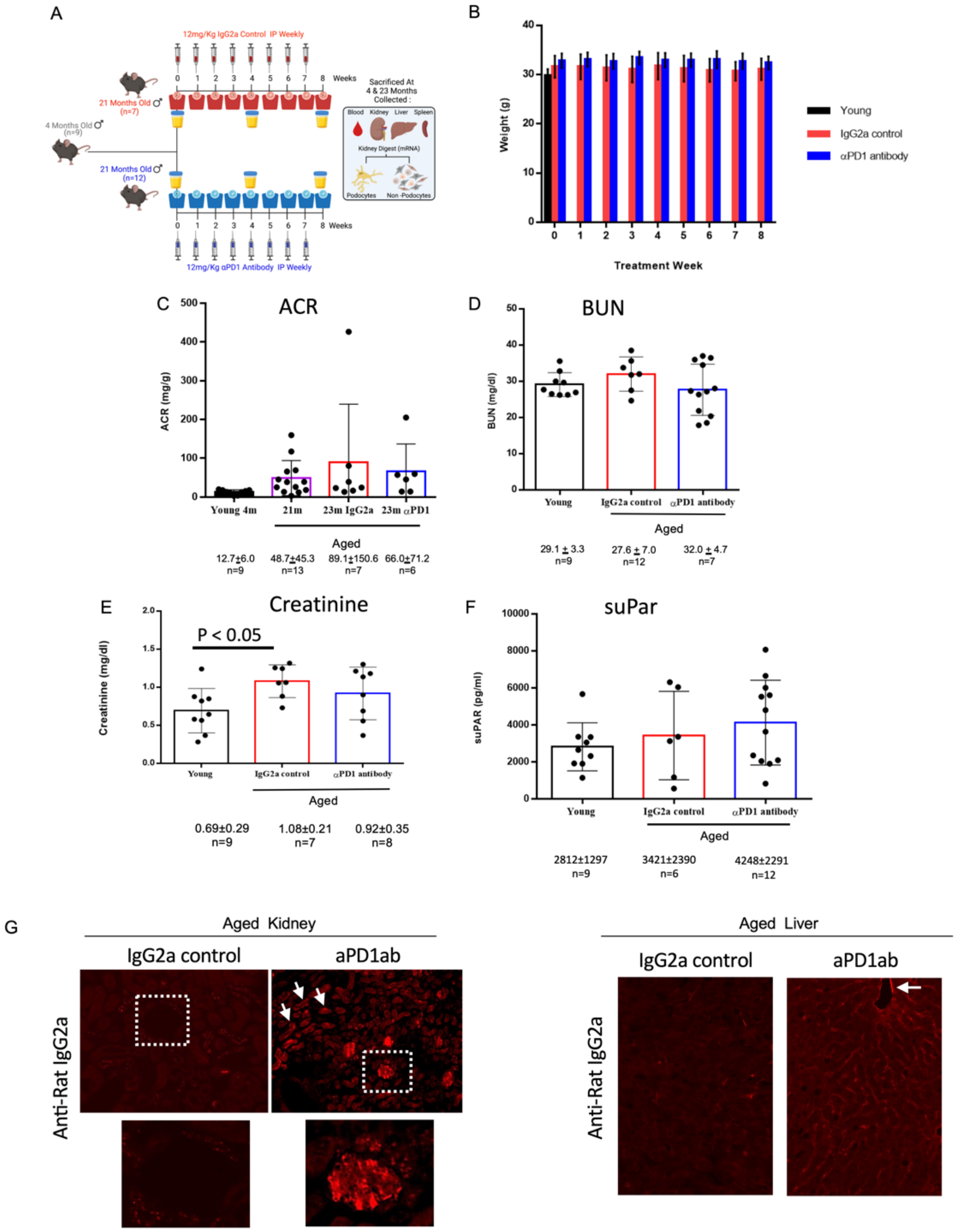

Supplemental Figure 1. Experimental Design and Physiological data for study animals. (**A**) Schematic of study design. 4-month-old mice comprised the young group. The aged group of 21-month-old mice were randomized to the control group which received 8 weekly intraperitoneal (IP) injections of rat IgG2a (control), or the treatment group which received 8 weekly IP injections of anti-PD1 antibody. (**B**) For the duration of the study, aged animals did not lose weight with weekly injections of either the IgG2a control (red bars) or anti-PD1 antibody (blue bars) and weights were similar to the young group (black bar). (**C-F**) For the duration of the study, kidney function parameters, ACR (**C**), BUN (**D**), creatinine (**E**) and plasma suPAR levels (**F**) were measured. Yet, none changed significantly between young (4 months), aged IgG2a control (23 months), or anti-PD1 antibody (23 months) injected mice, with the exception of creatinine levels between the young and the aged IgG2a-injected mice. (**G**) Immunofluorescence staining for rat IgG2a confirmed that the injected aPD1ab reached the glomerular (dashed box) and tubular (white arrows) epithelium and that the binding patterns was similar to the staining pattern observed for PD1 expression by immunofluorescence. Similarly, the distribution in the liver also reflected the PD1 expression pattern in the liver (white arrow). No signal was detected in the control IgG2a antibody-injected kidney and liver.

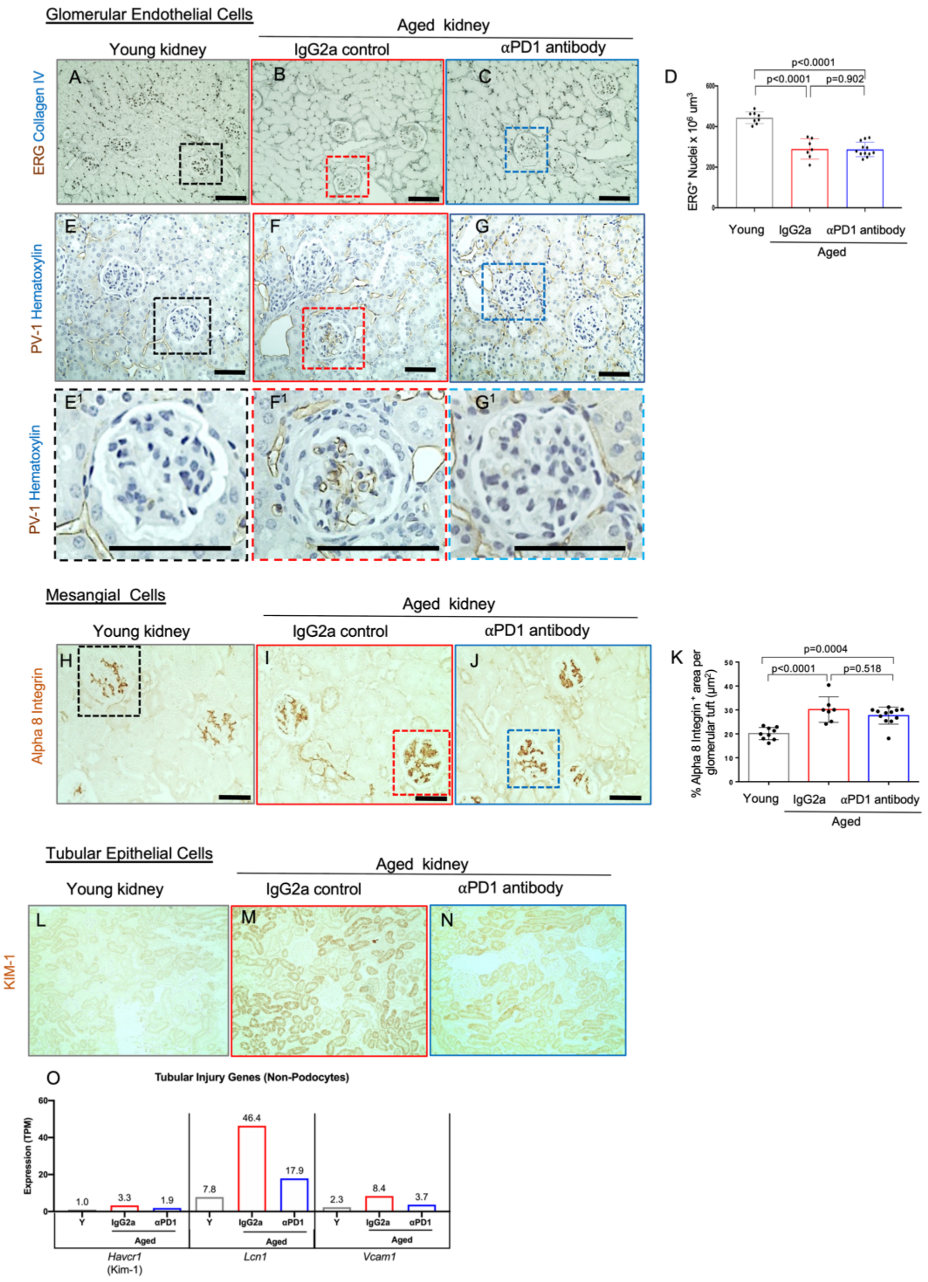

Supplemental Figure 2. Glomerular endothelial cell density/injury and mesangial cell density. (**A-D**) Representative images of double staining for the glomerular endothelial cell marker ERG (brown, nuclear) and collagen IV (blue, outlines glomeruli) and quantification thereof. Note that the age-dependent decrease in endothelial cell density does not change with aPD1ab treatment. (**E-G**) Representative images of immunoperoxidase staining for the fenestra diaphragm protein plasmalemmal vesicle associated protein-1 (PV-1) (brown) was not detected in glomerular endothelial cells of young mice, but was increased in aged IgG2a injected mice and decreased in aPD1ab injected mice. Superscripted images show higher magnification of the glomeruli highlighted by dashed boxes. (**H-K**) Representative images of immunoperoxidase staining for the mesangial cell marker alpha 8 Integrin (brown) and quantification thereof. Note increased staining in the aged IgG2a-injected mice that does not change with aPD1ab treatment. (**L-N**) Representative images of immunoperoxidase staining for the epithelial cell injury marker KIM-1 (brown). (**O**) Graph of the mRNA expression levels of the tubular injury genes *Havcr1*, *Lcn1* and *Vcam1*, which were increased in non-podocyte (mostly tubular epithelium) cell fractions of the aged IgG2a-injected mice and were lowered with aPD1ab treatment.

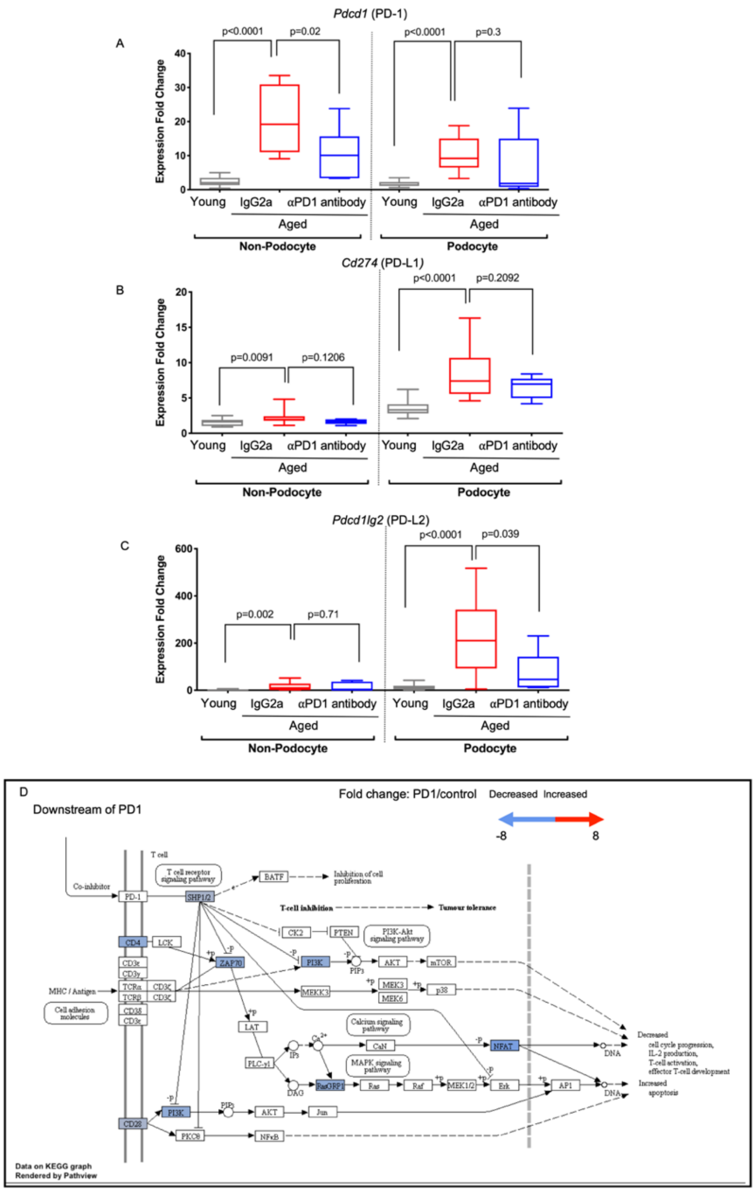

Supplemental Figure 3. Changes to the PD1 pathway in aged podocytes by anti-PD1 antibody. (**A-C**) mRNA expression measured by qRT-PCR for *Pdcd1* (*PD1*, **A**), *Cd274* (*PD-L1*, **B**) and *Pdcd1lg2* (*PD-L2*, **C**) in non-podocytes and podocyte cell fractions of the kidney. In the non-podocyte fraction, aPD1ab lowered *Pdcd1*, but did not change levels of *Cd274* or *Pdcd1lg2* compared to IgG2a-injected mice. In podocytes, aPD1ab injection appeared to lower *Pdcd1*, *Cd274* and *Pdcd1lg2*, compared to IgG2a-injected mice, but this did not reach statistical significance due to variation with the groups. (**D**) Gene expression data from the mRNA-seq experiment was mapped to PD1 target pathways in KEGG pathway database. Red rectangles denote up-regulated genes (aged/young expression ratio >1), blue rectangles denote down-regulated genes (aged/young expression ratio <1); grey rectangles denote genes with no significant change. Edges represent interactions between genes/proteins. "+p" denotes phosphorylation. Sharp arrows indicate positive, while dashed arrows indicate negative regulation. Scale in upper right shows fold change by color hue. Note that the panel shows an overall significant decrease in genes downstream of PD1 in aged podocytes from mice injected with aPD1ab compared to control aged-matched mice given IgG2a.

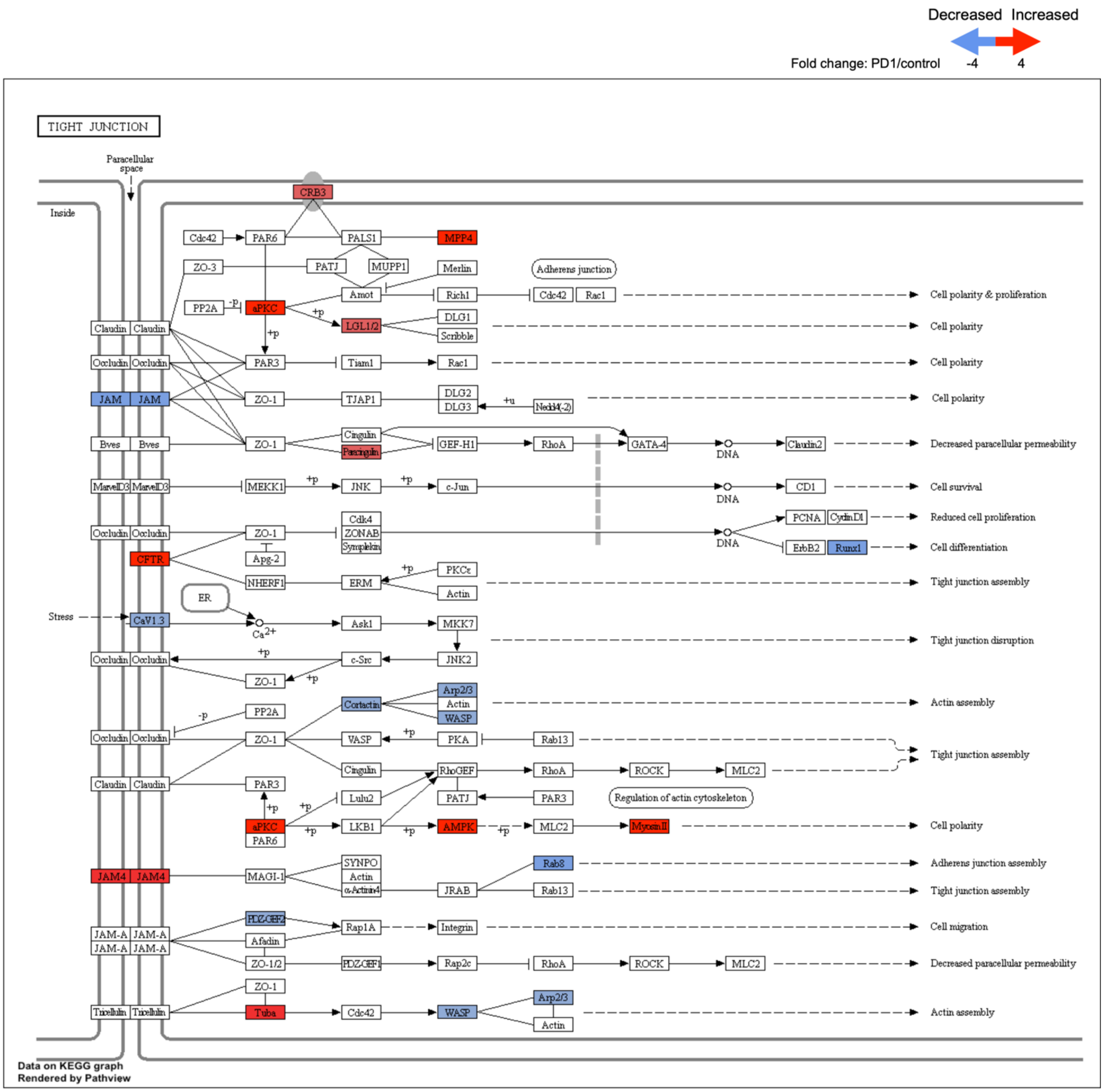

Supplemental Figure 4. Gene expression data mapped to the tight junction pathway in the KEGG pathway database. Gene expression data of the mRNA-seq experiment was mapped to tight junction pathways in the KEGG pathway database. Red rectangles denote up-regulated genes (aged/young expression ratio >1), blue rectangles denote down-regulated genes (aged/young expression ratio <1); grey rectangles denote genes with no significant change. Edges represent interactions between genes/proteins. "+p" denotes phosphorylation. Sharp arrows indicate positive, while dashed arrows indicate negative regulation. Scale in upper right shows fold change by color. The panel shows an overall significant increase in genes for tight junctions in aged podocytes from mice injected with aPD1ab compared to control aged-matched mice given IgG2a.

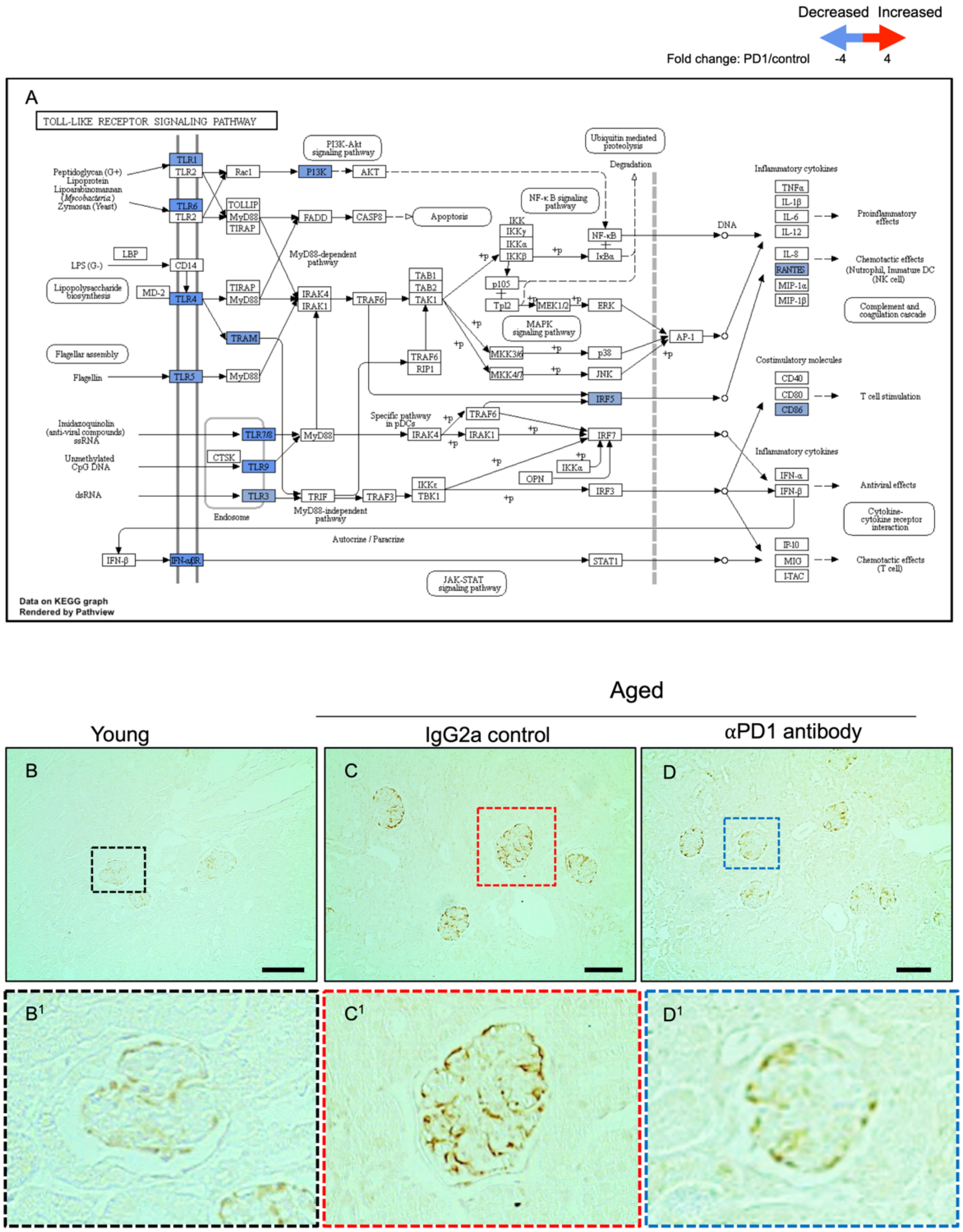

Supplemental Figure 5. Gene expression data mapped to Toll-Like Receptor Signaling Pathway in the KEGG pathway database. (**A**) Gene expression data from the mRNA-seq experiment was mapped to Toll-Like Receptor Signaling Pathway in the KEGG pathway database. Red rectangles denote up-regulated genes (aged/young expression ratio >1), blue rectangles denote down-regulated genes (aged/young expression ratio <1); grey rectangles denote genes with no significant change. Edges represent interactions between genes/proteins. “+p” denotes phosphorylation. Sharp arrows indicate positive, while dashed arrows indicate negative regulation. Scale in upper right shows fold change by color hue. The panel shows an overall significant decrease in genes for Toll-Like Receptor Signaling Pathway in aged podocytes from mice injected with aPD1ab compared to control aged-matched mice given IgG2a. (**B-D**) Representative images of immunoperoxidase staining for TLR4 (brown) show increased podocyte staining in aged IgG2a mice (**C**) compared to young mice (**B**), which is reduced in aged mice injected with aPD1ab confirming the changed observed in the mRNA (**D**). Panels with superscript show higher magnification of the glomeruli indicated by the dashed insets.

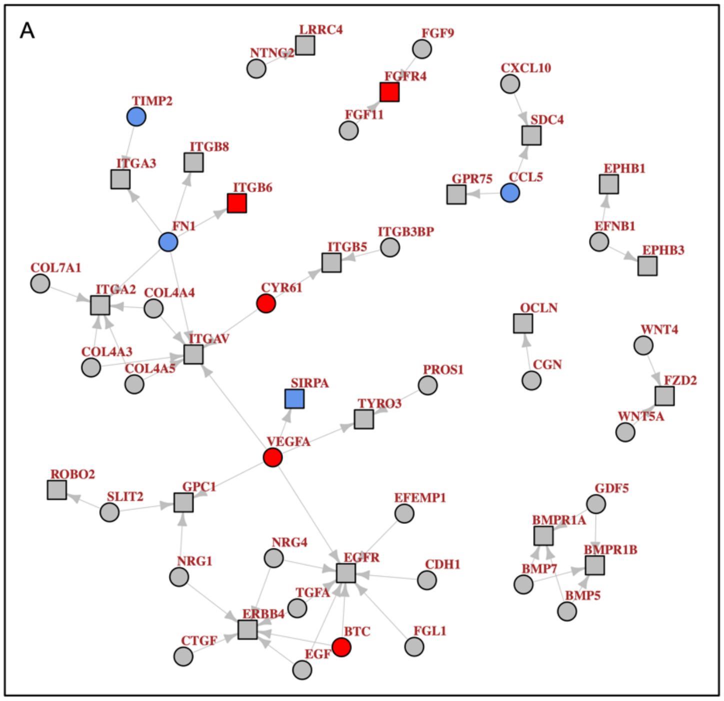

C

GENE	Fold change aged IgG2A /young	Fold change aged aPD1a /IgG2a
FGFR4	-8.5	+8.9
ITGB6	-4.8	+3.9
CYR61	-2.4	+3.5
VEGFa	-4.2	+3.4
BTC	-2.7	+3.7
TIMP2	1.2	-1.2
CCL5	1.5	-1.3
FN1	1.5	-1.7
SIRPA	1.6	-1.3

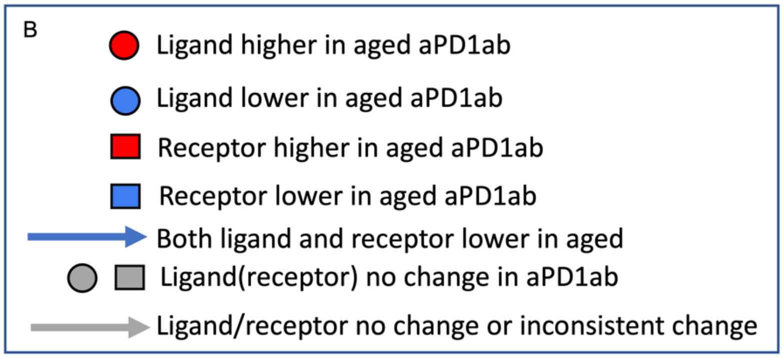

Supplemental Figure 6. Podocyte Ligand-Receptor Analysis. (**A-C**) Using the mRNA-seq data, ligands were mapped with their respective receptors based on their expression in the podocytes of aged IgG2a-injected mice compared to young podocytes. Data were overlaid for changes between the aged, aPD1ab-injected and the aged IgG2a-injected mice. The observed fold changes are summarized in (**C**).

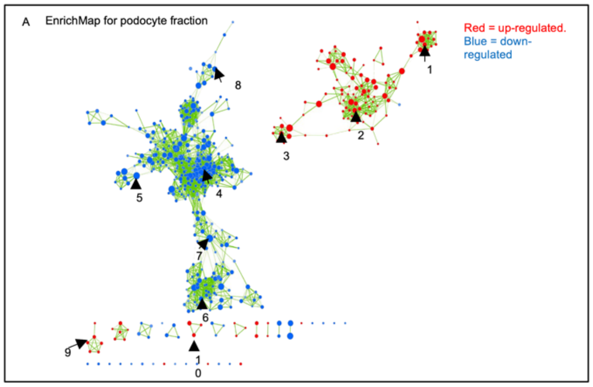

B

Node	Gene Ontology
1	Oxidative phosphorylation
2	Amino acid metabolism
3	Organic acid transport
4	T cell activation
5	Positive regulation of protein kinase activity
6	Mitotic nuclear division
7	DNA repair
8	Calcium ion transport
9	Mitochondrial translation
10	Glucose metabolism

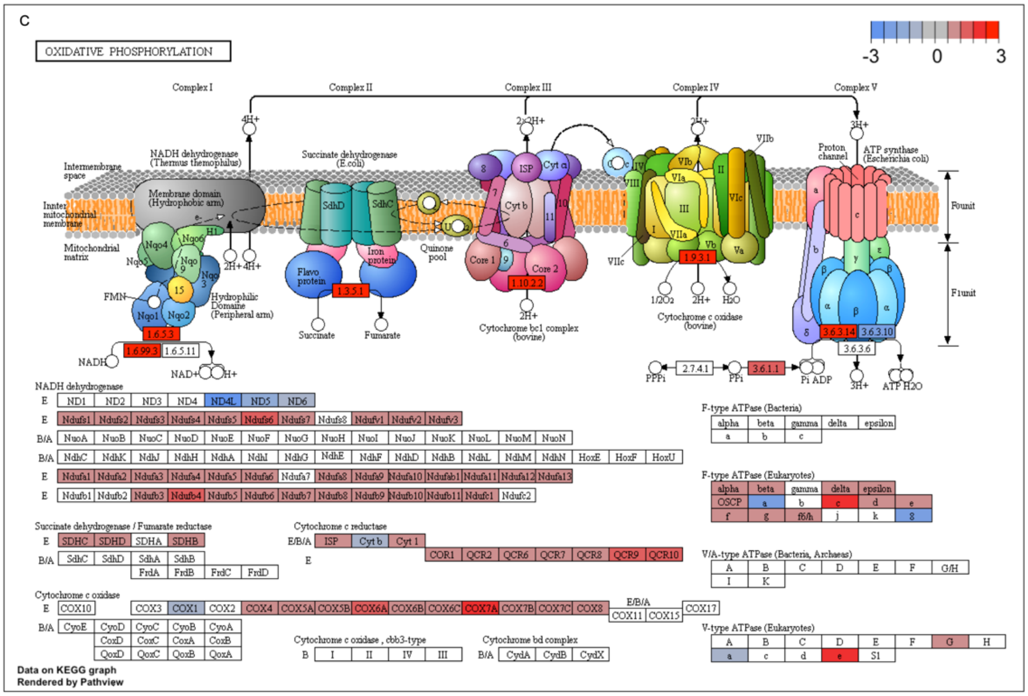

Supplemental Figure 7. Perturbed biological processes in podocyte aging and oxidative phosphorylation. (**A**) EnrichMap analysis of the mRNA-seq data, where each node represents a gene ontology term significantly enriched in genes up-regulated in aged podocytes (red nodes) or down-regulated in aged podocytes (blue nodes). The transparency of the node is proportional to enrichment p-value with more solid-colored nodes represent more significantly enriched GO terms. Node size represent gene set size so that GO terms with more genes have larger nodes. Two nodes are connected by an edge if they have overlapping genes. Edge transparency is proportional to the number of genes shared by two GO terms. Select GO terms from each cluster are labeled for each module. Overall, immune processes are up-regulated, while developmental and differentiation processes are down-regulated. Select nodes are numbered and their Gene Ontology terms are listed in the bottom right. (**B**) Table showing the names of the nodes for the pathways identified in (**A**). (**C**) Gene set enrichment analysis for Oxidative Phosphorylation mapped to the KEGG pathway database. Panel shows genes that are decreased (blue) and increased (red) in the oxidative phosphorylation pathway in aged mice injected with anti-PD1 antibody (aPD1ab) compared to control aged mice injected with the IgG2a. Scale in upper right shows with the degree of fold change indicated by color hue.

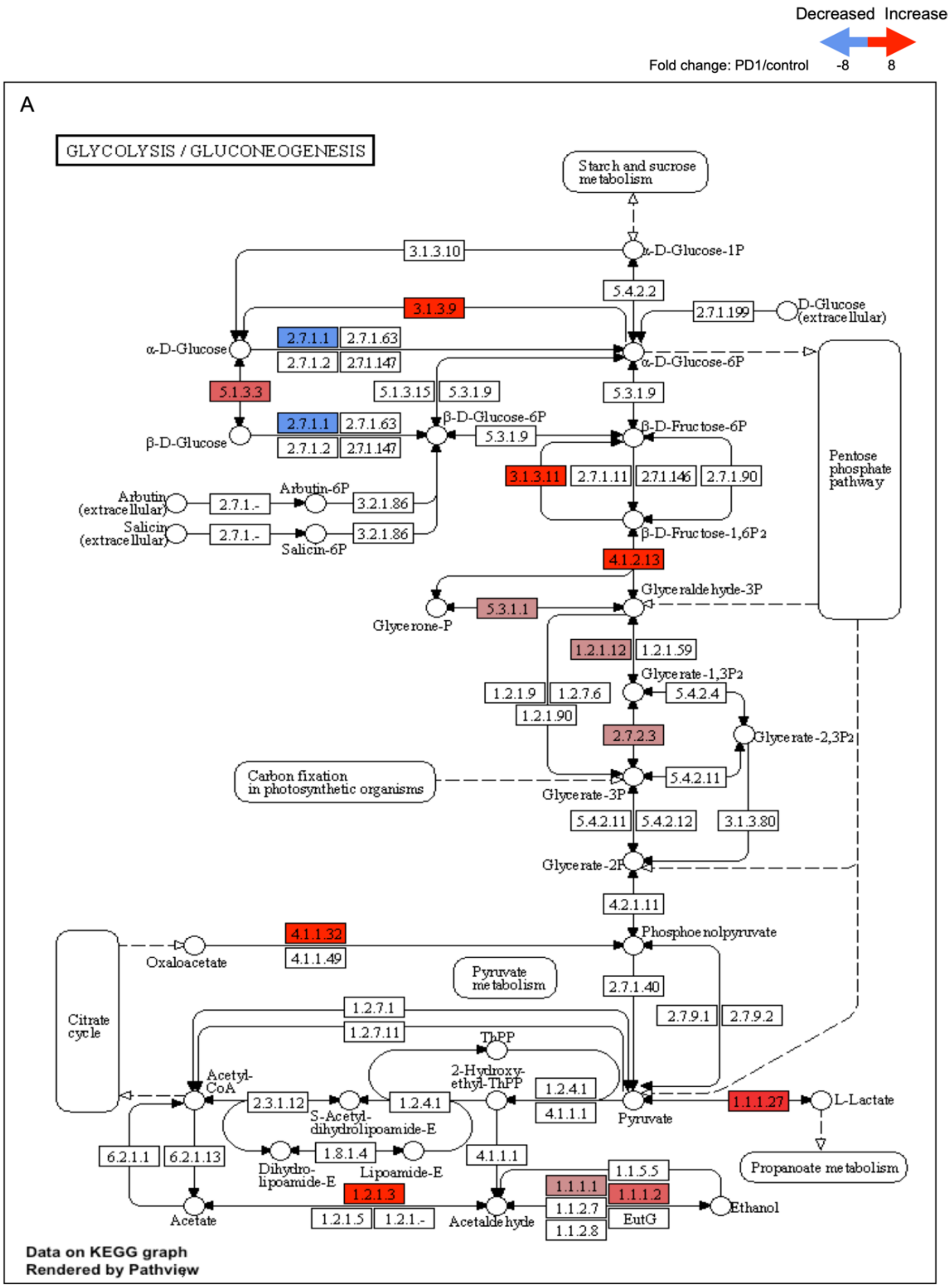

Supplemental Figure 8. Gene expression data mapped to the Glycolysis/Gluconeogenesis pathway in the KEGG pathway database. (A) Gene expression data was mapped to the Glycolysis/Gluconeogenesis Pathway in the KEGG pathway database. Red rectangles denote up-regulated genes (aged/young expression ratio >1), blue rectangles denote down-regulated genes (aged/young expression ratio <1); grey rectangles denote genes with no significant change. Edges represent interactions between genes/proteins. "+p" denotes phosphorylation. Sharp arrows indicate positive, while dashed arrows indicate negative regulation. Scale in upper right shows fold change by color hue. The panel shows an overall significant increase in genes for to the Glycolysis/Gluconeogenesis Pathway in aged podocytes from mice injected with aPD1ab compared to control aged-matched mice given IgG2a.

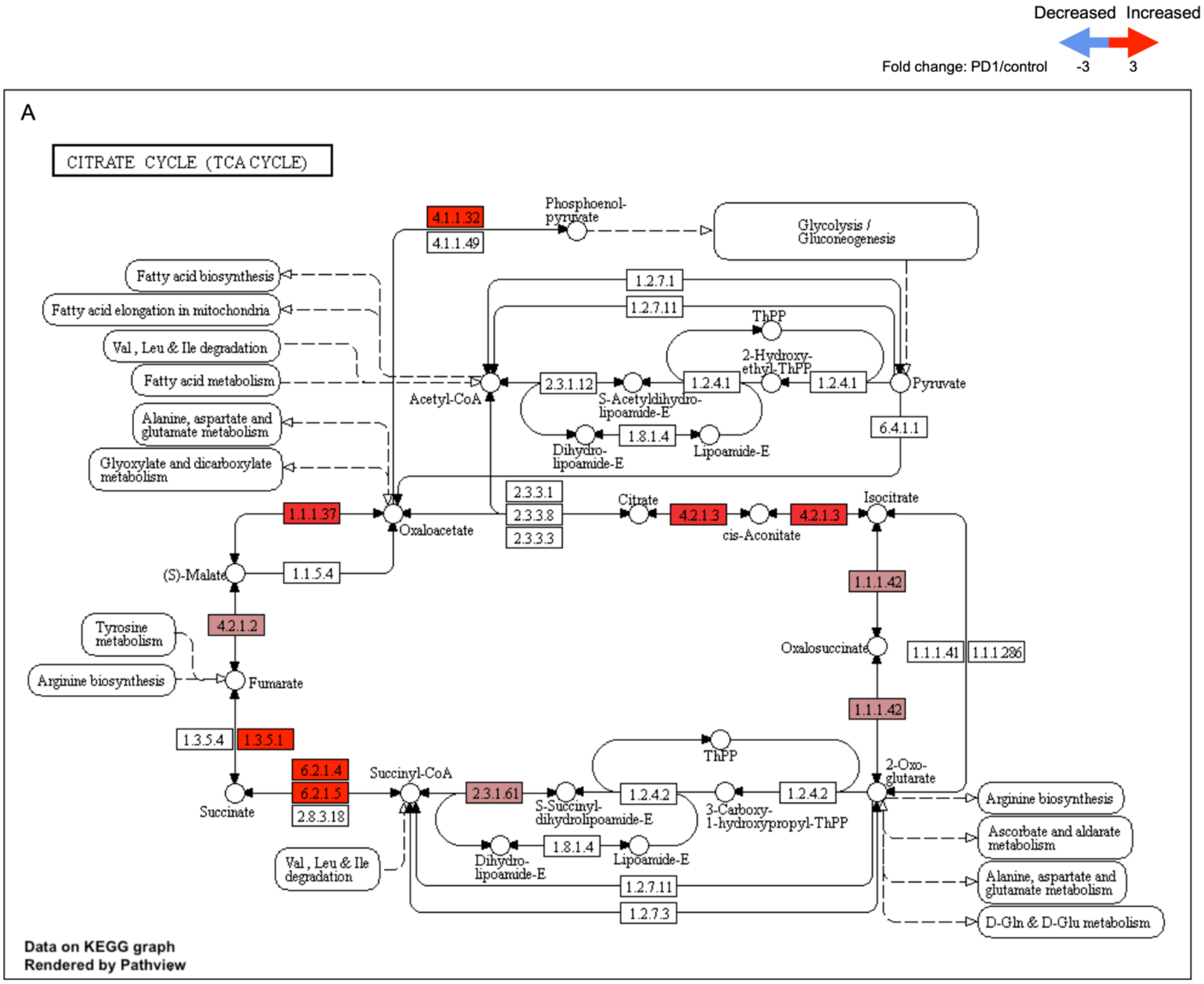

Supplemental Figure 9. Gene expression data mapped to the Citrate Cycle pathway in the KEGG pathway database. (**A**) Gene expression data was mapped to the Citrate Cycle Pathway in the KEGG pathway database. Red rectangles denote up-regulated genes (aged/young expression ratio >1), blue rectangles denote down-regulated genes (aged/young expression ratio <1); grey rectangles denote genes with no significant change. Edges represent interactions between genes/proteins. “+p” denotes phosphorylation. Sharp arrows indicate positive, while dashed arrows indicate negative regulation. Scale in upper right shows fold change by color hue. The panel shows an overall significant increase in genes for to the Citrate Cycle Pathway in aged podocytes from mice injected with aPD1ab compared to control aged-matched mice given IgG2a.

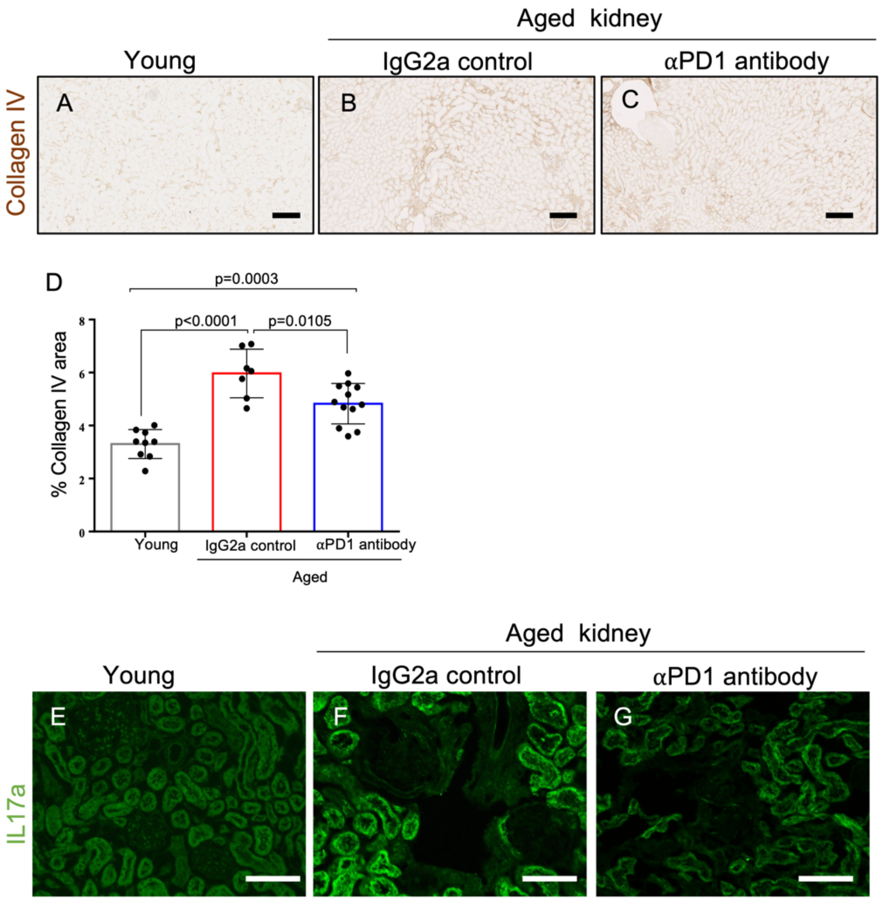

Supplemental Figure 10. Tubulointerstitial Changes with aPD1ab injection. (**A-D**). Representative images of Collagen IV immunoperoxidase staining (brown) and their quantification thereof. Note that compared to young kidneys, Collagen IV staining was higher in the interstitium of aged IgG2a-injected mice, which was decreased by aPD1ab injection. (**E-G**) Representative images of Interleukin 17a (IL17a) immunofluorescence staining (green). Compared to young kidneys (**E**), IL17a staining was higher in the tubular epithelial cells in aged IgG2a-injected mice (**F**) and was decreased by aPD1ab injection (**G**).

Supplemental Videos:

Supplemental Video 1. Z-Projection of FLARE-labeled glomeruli from young mice. Representative z-projection of images from expansion microscopy of FLARE-labeled glomeruli in young mice showing the glomerular basement membrane (pink).

Supplemental Video 2. Z-Projection of FLARE-labeled glomeruli from aged mice. Representative z-projection of images from expansion microscopy of FLARE-labeled glomeruli in aged, IgG2a-injected mice showing the glomerular basement membrane (pink).

Supplemental Video 3. Z-Projection of FLARE-labeled glomeruli from aged mice. Representative z-projection of images from expansion microscopy of FLARE-labeled glomeruli in aged, aPD1ab-injected mice with the showing the glomerular basement membrane (pink).

Supplemental Table 1: Patient Characteristics Receiving Immune Checkpoint Inhibitors (ICPI).

Age (years)	Sex	Malignancy	ICPI	Baseline SCreat (mg/dl)	SCreat at Biopsy (mg/dl)	Proteinuria	Pathological Diagnosis	EM
52	F	Ovarian	Nivolumab	1.6	3.1	Negative (UA)	ATIN	Well preserved foot processes
59	F	H&N SCC	Pembrolizumab Nivolumab	1.1	1.82	Negative (UA)	CATIN	Focal foot process effacement (<20%)
79	M	Melanoma	Pembrolizumab	0.89	2.46	Negative (UA)	Acute tubular injury	Focal foot process effacement (<5%)
91	M	Melanoma	Pembrolizumab	2.29	3.45	14g/g 9.8 Alb/Cr	ATIN	No EM
79	M	SCC scalp	Cemiplimab	1.1	1.1	Not done	ATIN	Swollen podocytes (bloodless glomeruli)
69	M	Melanoma	Nivolumab Pembrolizumab	1.14	3.53	1.4 (Pr/Cr)	ATIN	Well preserved foot processes
79	F	SCC	Pembrolizumab	<2.0	4.4	1+ (UA)	ATIN	Segmental foot process effacement (40-50%)
70	F	Lung adeno	Atezolizumab	1.2	1.7	Negative (UA)	PTN	Focal foot process effacement (<10%)
65	M	Pancoast tumor	Nivolumab	1.1	3.6	0.06 (Alb/Cr)	PTN	Well preserved foot processes
81	M	Melanoma	Nivolumab	1.02	1.94	Not available	CATIN	No EM

Abbreviations:
M = male; F=Female; SCreat = Serum creatinine; EM = electron microscopy, H&N = head and neck, SCC = small cell carcinoma; UA = urine analysis by dipstix, Alb/Cr = Albumin/Creatinine ratio, Pr/Cr = Protein/Creatinine ratio, ATIN acute tubulointerstitial nephritis; CATIN chronic and active tubulointerstitial nephritis; PTN patchy tubulointerstitial nephritis

Supplemental Table 2: List of Antibodies.

Primary antibody	Antibody to identify	Raised in	Working dilution	Antigen retrieval buffer	Source/Catalog Number
**p57**	Podocytes	Rabbit	1:800	EDTA buffer pH6	Santa Cruz Biotechnology, Santa Cruz, CA, USA sc8298
**Collagen type IV**	Glomerular injury	Rabbit	1:200	EDTA buffer pH6	Southern Biotechnology, Birmingham, AL, USA 1430-01
**Synaptopodin**	Actin-associated protein in renal podocytes	Mouse	1:500	EDTA buffer pH8	Fitzgerald Industries International. Inc., Concord, MA RDI-PRO65194
**Nephrin**	Slit diaphragm of podocytes	Guinea pig	1:1500	EDTA buffer pH6	Fitzgerald Industries International. Inc., Concord, MA RDI-PROGPN2
**Podocin**	Podocytes	Rabbit	1:4000	EDTA buffer pH6	Abcam, Cambridge, MA, USA Ab50339
**Nephrin (Y17-R)**	Extracellular Domain	Rabbit	1:100	MACS	MyBiosource.com MBA684100
**Nephrin (G17-H)**	Extracellular Domain	Rabbit	1:100	MACS	MyBiosource.com MBS684143
**Desmin**	Podocyte injury/stress marker	Rabbit	1:1000	Citrate buffer pH 6	Abcam, Cambridge, MA, USA ab15200
**VEGF-A**	Podocyte synthesis	Rabbit	1:200	Citrate buffer pH 6	Abcam, Cambridge, MA, USA ab52917
**IgG2a heavy chain**	Presence of injected anti-PD-1 drug	Rat	1:200	Citrate buffer pH 6	Abcam, Cambridge, MA, USA ab172333
**PD1**	Programmed cell death	Rabbit	1:100	Citrate buffer pH 6	Sino Biological, Wayne, PA, USA 50124-RP02
**PD-L1**	Programmed cell death	Rabbit	1:200	Citrate buffer pH 6	Cell Signaling, Danvers, MA, USA 64988S
**GRP94**	Endoplasmic reticulum stress	Rabbit	1:200	Citrate buffer pH 6	Thermo Fisher Scientific, Waltham, MA, USA MA3-016
**Cleaved caspase 3 antibody**	Apoptosis	Rabbit	1:200	Citrate buffer pH 6	Cell Signaling, Danvers, MA, USA 9579S
**LC3**	Autophagy	Rabbit	1:200	Citrate buffer pH 6	Sigma-Aldrich, St. Louis, MO, USA L8918

**CD31**	Endothelial cells	Rat	1:200	Citrate buffer pH 6	Dianova, Hamburg, Germany DIA-310
**PV1**	Microvascular endothelial cells	Rabbit	1:100	EDTA buffer pH6	(PV1) antibody (1:100, BD Bioscience, San Jose, CA 550563
**α8 integrin**	Mesangial cells	Goat	1:100	Citrate buffer pH 6	R&D Systems Inc., Minneapolis, MN, USA BAF4076
**CD45**	all nucleated hematopoietic cells (immune cells)	Rabbit	1:100	None	Novus Biological, Littleton, CO, USA NB100
**Lotus Tetragonolobus Lectin/LTL**	Proximal tubular epithelial cells	Tetragonolobus purpureus	1:500	Citrate buffer pH 6	Vector Labs, Burlingame, CA, USA B-1325-2
**Megalin/LRP2**	Proximal tubular epithelial cells	Rabbit	1:1000	Citrate buffer pH 6	Sino Biological, Wayne, PA, USA 106515-T08
**p19**	Senescence	Rabbit	1:800	EDTA buffer pH8	Lifespan Biosciences, Seattle, WA, USA
**p16INK4a-N-terminal**	Senescence	Rabbit	1:1000	Citrate buffer pH 6	Abcam, Cambridge, MA, USA ab189034
**SA-β-galactosidase**	Senescence	Chemical component	1:1000	None	Cell Signaling, Danvers, MA, USA 9860S
**PAX8**	Parietal epithelial cells	Rabbit	1:500	EDTA buffer pH6	Protein Tech Group, Chicago, IL, USA 10336-1-AP
**CD44**	"Activated" parietal epithelial cells	Rat	1:50	Citrate buffer pH 6	BD Biosciences, San Jose, CA, USA 553131
**CD74**	"Activated" parietal epithelial cells	Rabbit	1:100	Citrate buffer pH 6	BD Biosciences, San Jose, CA),USA 555317
**pERK**	"Activated" parietal epithelial cells	Rabbit	1:100	Citrate buffer pH 7	Cell Signaling Technology, Beverly, MA, USA 9101S
**pS6RP**	To determine activation of the mammalian target of rapamycin (mTOR) pathway	Rabbit	1:100	Citrate buffer pH 6	Cell Signaling Technology, Beverly, MA, USA 2217S
**KIM1/TIM1**	Upregulated on the surfaces of kidney epithelia	Rabbit	1:200	Citrate buffer pH6	Novus Biological, LLC, CO 80112, USA NBP1

REFERENCES

83. Dutta S, and Sengupta P. Men and mice: Relating their ages. *Life Sciences.* 2016;152:244-8.
84. Fox JG. *The Mouse in Biomedical Research: Normative Biology, Husbandry, and Models.* Amsterdam ; Boston: Elsevier; 2007.
85. Marshall CB, Krofft RD, Pippin JW, and Shankland SJ. CDK inhibitor p21 is prosurvival in adriamycin-induced podocyte injury, in vitro and in vivo. *Am J Physiol Renal Physiol.* 2010;298(5):F1140-51.
86. Ohse T, Vaughan MR, Kopp JB, Krofft RD, Marshall CB, Chang AM, et al. De novo expression of podocyte proteins in parietal epithelial cells during experimental glomerular disease. *American Journal of Physiology- Renal Physiology.* 2010;298(3):F702-11.
87. Zhang J, Pippin JW, Krofft RD, Naito S, Liu Z, and Shankland SJ. Podocyte Repopulation by Renal Progenitor Cells Following Glucocorticoids Treatment in Experimental FSGS. *Am J Physiol Renal Physiol.* 2013;304(11):F1375-89.
88. Zhang J, Pippin JW, Vaughan MR, Krofft RD, Taniguchi Y, Romagnani P, et al. Retinoids Augment the Expression of Podocyte Proteins by Glomerular Parietal Epithelial Cells in Experimental Glomerular Disease. *Nephron Exp Nephrol.* 2012;121(1-2):e23-e37.
89. Kaverina NV, Eng DG, Miner JH, Pippin JW, and Shankland SJ. Parietal epithelial cell differentiation to a podocyte fate in the aged mouse kidney. *Aging (Albany NY).* 2020;12(17):17601-24.
90. Kaverina NV, Eng DG, Schneider RR, Pippin JW, and Shankland SJ. Partial podocyte replenishment in experimental FSGS derives from nonpodocyte sources. *Am J Physiol Renal Physiol.* 2016;310(11):F1397-413.
91. Cui A, Hu Z, Han Y, Yang Y, and Li Y. Optimized Analysis of In Vivo and In Vitro Hepatic Steatosis. *J Vis Exp.* 2017(121).
92. Zhu LM, Song YF, and Yu J. [Histology, cytochemistry and ultrastructure of APUD cells in the neonatal rabbit lung]. *Hua Xi Yi Ke Da Xue Xue Bao.* 1987;18(4):339-42.
93. Heeringa SF, Vlangos CN, Chernin G, Hinkes B, Gbadegesin R, Liu J, et al. Thirteen novel NPHS1 mutations in a large cohort of children with congenital nephrotic syndrome. *Nephrol Dial Transplant.* 2008;23(11):3527-33.
94. Liao Y, Smyth GK, and Shi W. The Subread aligner: fast, accurate and scalable read mapping by seed-and-vote. *Nucleic Acids Res.* 2013;41(10):e108.
95. Anders S, Pyl PT, and Huber W. HTSeq--a Python framework to work with high-throughput sequencing data. *Bioinformatics.* 2015;31(2):166-9.
96. Anders S, and Huber W. Differential expression analysis for sequence count data. *Genome Biol.* 2010;11(10):R106.
97. Subramanian A, Tamayo P, Mootha VK, Mukherjee S, Ebert BL, Gillette MA, et al. Gene set enrichment analysis: a knowledge-based approach for interpreting genome-wide expression profiles. *Proc Natl Acad Sci U S A.* 2005;102(43):15545-50.
98. Xiao Y, Hsiao TH, Suresh U, Chen HI, Wu X, Wolf SE, et al. A novel significance score for gene selection and ranking. *Bioinformatics.* 2014;30(6):801-7.
99. Alexa A, Rahnenfuhrer J, and Lengauer T. Improved scoring of functional groups from gene expression data by decorrelating GO graph structure. *Bioinformatics.* 2006;22(13):1600-7.
100. Kanehisa M, and Goto S. KEGG: kyoto encyclopedia of genes and genomes. *Nucleic Acids Res.* 2000;28(1):27-30.
101. Luo W, and Brouwer C. Pathview: an R/Bioconductor package for pathway-based data integration and visualization. *Bioinformatics.* 2013;29(14):1830-1.
102. Schaub JA, O'Connor CL, Shi J, Wiggins RC, Shedden K, Hodgin JB, et al. Quantitative morphometrics reveals glomerular changes in patients with infrequent segmentally sclerosed glomeruli. *J Clin Pathol.* 2021.

103. Lee HJ, Donati A, Feliers D, Sun Y, Ding Y, Madesh M, et al. Chloride channel accessory 1 integrates chloride channel activity and mTORC1 in aging-related kidney injury. *Aging Cell.* 2021;20(7):e13407.
